# The Prevalence of Malaria and Bacteremia Co-Infections among Febrile Patients: A Systematic Review and Meta-Analysis

**DOI:** 10.3390/tropicalmed7090243

**Published:** 2022-09-13

**Authors:** Polrat Wilairatana, Wanida Mala, Frederick Ramirez Masangkay, Kwuntida Uthaisar Kotepui, Manas Kotepui

**Affiliations:** 1 Department of Clinical Tropical Medicine, Faculty of Tropical Medicine, Mahidol University, Bangkok 10400, Thailand; 2 Medical Technology, School of Allied Health Sciences, Walailak University, Nakhon Si Thammarat 80160, Thailand; 3 Center of Excellence Research for Melioidosis and Microorganisms, Walailak University, Nakhon Si Thammarat 80160, Thailand; 4 Department of Medical Technology, Institute of Arts and Sciences, Far Eastern University–Manila, Manila 1008, Philippines

**Keywords:** malaria, bacteremia, co-infection, concomitant infection, meta-analysis

## Abstract

Comprehensive data on the relative contribution of bacteremia to malaria outcomes in a large number of participants are lacking. Therefore, we collated data on the co-existence of malaria and bacteremia in the literature to provide evidence-based information for future studies investigating the clinical significance of this co-infection. The study protocol was registered at PROSPERO (ID: CRD42021287971). Relevant studies were identified from PubMed, Web of Science, and Scopus. The pooled prevalence of (1) co-existent malaria and bacteremia among febrile patients, (2) the pooled prevalence of bacteremia among patients with malaria, (3) the probability of co-infection, and (4) the pooled prevalence of deaths were estimated by the random-effects model. Fifty-one studies involving 1583 cases of co-infection were included in the analyses. Typhoidal *Salmonella* spp. and *Staphylococcus aureus* were the most common Gram-negative and Gram-positive bacteria, respectively. The prevalence of co-existent malaria and bacteremia among febrile patients was 1.9% (95% confidence interval (CI) = 1.5–2.2%, *I*^2^ = 96.64%, 31 studies). The prevalence of bacteremia among patients with malaria was 7.6% (95% CI = 6.7–8.7%, and *I*^2^ = 96.68%, 43 studies). Co-infection by malaria and bacteremia did not occur by chance (*p* = 0.024, odds ratio = 0.64, 95% CI = 0.43–0.94, and *I*^2^ = 95.7%, 29 studies). The pooled prevalence of deaths among patients with co-infection was 15.0% (95% CI = 8.0–23.0%, *I*^2^ = 75.23%, 8 studies). On the basis of this study, we conclude that although the prevalence of co-infection was low, patients with malaria appear at greater risk of bacteremia and death.

## 1. Introduction

Malaria has long been a major cause of mortality in children younger than 5 years in Africa [1]. In Africa, the transmission intensity of malaria is extremely high, and malaria treatment is presumptively provided to patients who present with fever [2]. In recent years, more rapid diagnostic tests (RDTs) are being used, and even Points of Care Tests (POCTs) are being implemented in rural areas in order to improve malaria diagnoses apart from microscopy in endemic areas, particularly areas with unavailable electrical facilities for microscopy. This has encouraged the over-diagnosis and over-treatment of malaria in malaria-endemic countries, which can lead to poor outcomes and prognoses. Malaria and other acute febrile illnesses (AFIs) can be difficult to distinguish in a differential diagnosis in resource-limited settings because of their overlapping clinical signs. Concomitant malaria and AFIs, such as malaria with co-existent dengue [3,4], chikungunya [5], leptospirosis [6], visceral leishmaniasis [7], and scrub typhus [8], were well described in previous systematic reviews. Therefore, co-infection by pathogens could lead to the over-prescription of antimicrobial agents, which are frequently recognized as a major driver of drug resistance [9].

After bacteremia, the common causes of febrile illness in Africa include typhoidal and non-typhoidal *Salmonella*, *Staphylococcus aureus*, *Streptococcus pneumoniae*, *Klebsiella pneumoniae*, *Haemophilus influenzae*, and *Escherichia coli* [10,11,12,13,14,15]. Bacteremia and malaria are difficult to differentiate clinically in resource-limited settings. Because malaria RDTs are increasingly available in these settings, fever caused by malaria may easily be excluded [16]. However, causes of non-malaria febrile illness such as bacteremia remain problematic. Therefore, the management of bacteremia in resource-limited settings relies mainly on clinical diagnosis and consequently the impropriate treatment of patients with antimicrobial agents [17].

Several studies have documented co-existent malaria and types of bacteremia such as *Salmonella* infection [18,19,20,21] or pneumonia [22,23]. Our recent systematic review reported a pooled prevalence of typhoidal/non-typhoidal *Salmonella* (NTS) and malaria co-infection among febrile patients of 14% [24]. In addition, malaria was associated with typhoidal/NTS in children aged < 15 years [24]. A previous systematic review investigating co-existent malaria and bacteremia based on a limited number of studies revealed a co-infection rate of 5.58%, and 24.1% of co-infected patients died [25]. However, the review was subject to methodological limitations, including a limited number of studies, high heterogeneity, and publication bias. Clinicians in low-income countries may rely on empirical treatment guided by World Health Organization (WHO); it might increase the risk of poor clinical outcomes and antimicrobial resistance in the study settings [26]. Therefore, we collated data on the co-existence of malaria and bacteremia from the literature to provide evidence-based information for a better understanding of co-infection to improve clinical management and outcomes and guide further studies investigating the clinical significance of this co-infection.

## 2. Methods

### 2.1. Protocol and Registration

The reporting of this systematic review and meta-analysis followed the Preferred Reporting Items for Systematic Reviews and Meta-Analyses statement [27]. The study protocol was registered at PROSPERO (ID: CRD42021287971).

### 2.2. Eligibility Criteria and Search Strategy

Studies that reported patients with co-existent malaria and bacteremia were included. Meanwhile, case reports, case series, letters to the editor, short reports, commentaries, studies without available full text, and studies with incomplete data for extraction were excluded. Combinations of search terms “(malaria OR plasmodium) AND (bacteremia)” were used to identify the potentially relevant studies in PubMed, Web of Science, and Scopus (Table S1). The searches were performed between 10 September 2021 and 13 September 2021. The search terms “malaria” and “bacteremia” were checked with medical subject heading terms in the National Library of Medicine to ensure that search terms were appropriately and generally used in the published literature.

### 2.3. Study Selection and Data Extraction

Two authors (MK and WM) were responsible for study selection and data extraction. Disagreement between the two authors was resolved by discussion to reach a consensus. If agreement could not be achieved, then a third author (PW) was responsible for the final decision on study selection. The following data were extracted and entered in the pilot-standardized spreadsheet: the name of the first author, the publication year, the study sites and years, the study design, the types of participants, the number of participants, the age range, sex, the number of cases of malaria and bacteremia co-infection, the number of cases of malaria, the number of cases of bacteremia, the clinical outcomes of co-infection, the diagnostic methods for malaria, and the diagnostic methods for bacteremia.

### 2.4. Quality of the Included Studies

The quality of the included studies was assessed using the critical appraisal tools of the Strengthening the Reporting of Observational Studies in Epidemiology (STROBE) [28] by two authors (MK, WM). Studies with scores of at least 75% of the maximum were deemed high-quality studies, whereas studies with scores less than 75% of the maximum were considered moderate- or low-quality studies.

### 2.5. Data Syntheses

The pooled prevalence of (1) co-existent malaria and bacteremia among febrile patients, (2) the pooled prevalence of bacteremia among patients with malaria, and (3) the pooled prevalence of deaths among co-infection were estimated by the random-effects model. The heterogeneity of the prevalence of co-infection was assessed using Cochrane’s Q with *p* < 0.05 or the *I*^2^ statistic, for which values exceeding 50% indicated substantial heterogeneity. Subgroup analysis of the participants’ characteristics was performed if the prevalence of co-infection among the included studies was heterogenous. Publication bias was assessed using the funnel plot and Egger’s test. If publication bias was identified, the trim and fill method was used in the sensitivity analysis to estimate the pooled prevalence of malaria and bacteremia co-infection. Quantitative analysis was performed using Stata version 14.2 (StataCorp LLC, College Station, TX, USA).

## 3. Results

### 3.1. Search Results

**Figure 1:**
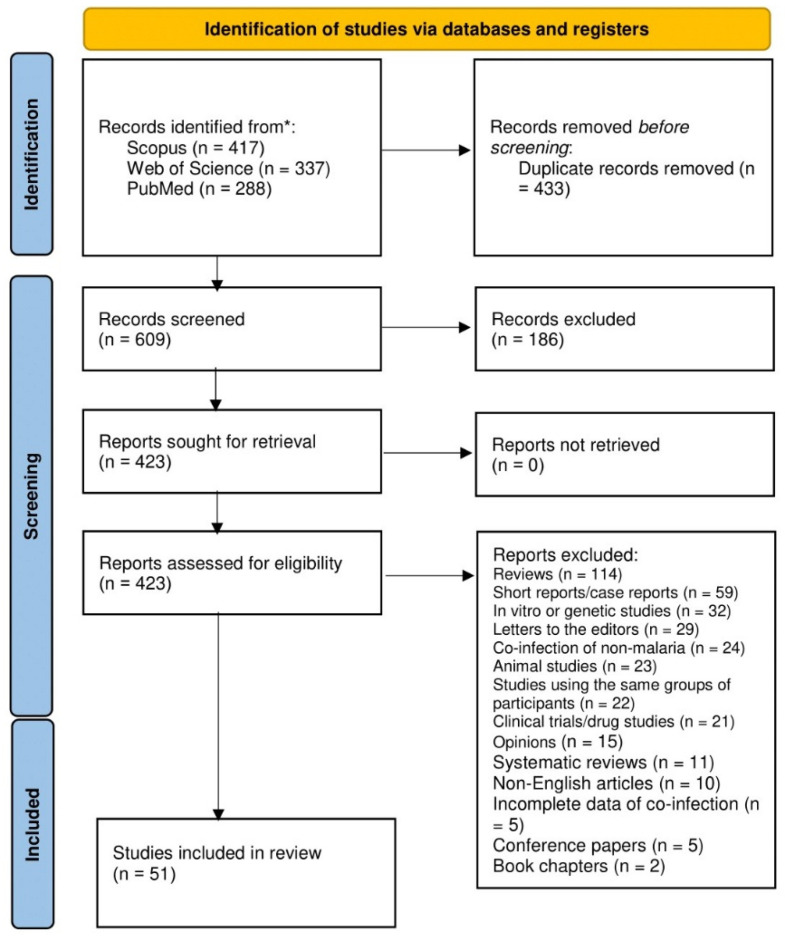
Study flow diagram.

In total, 1042 studies in 1973–2021 were identified from 3 databases databases (PubMed, 288; Scopus, 417; Web of Science, 337). After study selection was performed, 51 studies in 1993–2021 [12,15,26,29,30,31,32,33,34,35,36,37,38,39,40,41,42,43,44,45,46,47,48,49,50,51,52,53,54,55,56,57,58,59,60,61,62,63,64,65,66,67,68,69,70,71,72,73,74,75,76] met the eligibility criteria, and they were included in the qualitative and quantitatively syntheses (Figure 1).

### 3.2. Characteristics of the Included Studies

**Figure 2:**
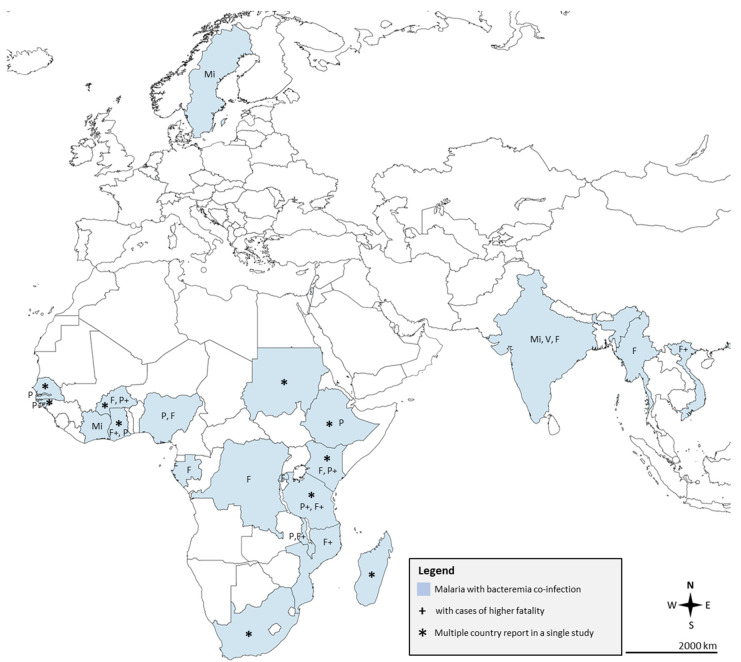
Global distribution of malaria with bacteremia co-infection. Abbreviations: **P**, *Plasmodium* spp.; **F**, *Plasmodium falciparum*; **V**, *Plasmodium vivax*; and **Mi**, Mixed *Plasmodium* spp. (includes *Plasmodium ovale* or *Plasmodium malariae*, or both in some included studies). Note: Declaration of either *Plasmodium* spp. or Malaria in the included studies was designated as *Plasmodium* spp. for the causative agent. Map was retrieved and modified by the authors from https://mapchart.net/world.html. Authors are allowed to use, edit, and modify any map created with mapchart.net for publication freely by adding the reference to mapchart.net.

Most of the included studies were conducted in Africa (44/51, 86.3%), Asia (6/51, 11.8%), and Europe (1/51, 1.96%). Most of the included studies were prospective observational (26/51, 51.0%), cross-sectional (15/51, 29.4%), case-control (5/51, 9.8%), and retrospective studies (5/51, 9.8%). The details of the included studies are presented in Table S2. The global distribution of malaria with bacteremia co-infection is shown in Figure 2.

### 3.3. Quality of the Included Studies

Among prospective observational studies, 15 studies (78.9%) were high-quality, and 4 studies were moderate-/low-quality. Among cross-sectional studies, 13 studies (86.7%) were high-quality, and 2 studies were moderate-/low-quality. Seven prospective cohort studies and 4 retrospective studies were high-quality. Among case-control studies, 3 studies (50%) were high-quality, whereas the others were moderate-/low-quality (Table S3). Moderate- and low-quality studies were retained for qualitative syntheses.

### 3.4. Co-Infection Types

Among 1583 cases from 51 studies, most cases of co-infection involved *P*. *falciparum* and Gram-negative bacteria (648 cases, 35%), *P*. *falciparum* and bacteremia (unidentified bacteria, 391 cases, 21.1%), and *P*. *falciparum* and Gram-positive bacteria (176 cases, 9.5%). Among cases of *P*. *falciparum* and Gram-negative bacterium co-infection, typhoidal *Salmonella* was the most common cause of bacteremia (414/648, 63.9%), whereas among cases of *P*. *falciparum* and Gram-positive bacterium co-infection, *Staphylococcus aureus* (79/176, 44.9%) was the most common infecting bacterium. Meanwhile, other characteristics of malaria and bacteremia co-infection are presented in Table 1.

### 3.5. Prevalence of Malaria and Bacteremia Co-Infection

**Figure 3:**
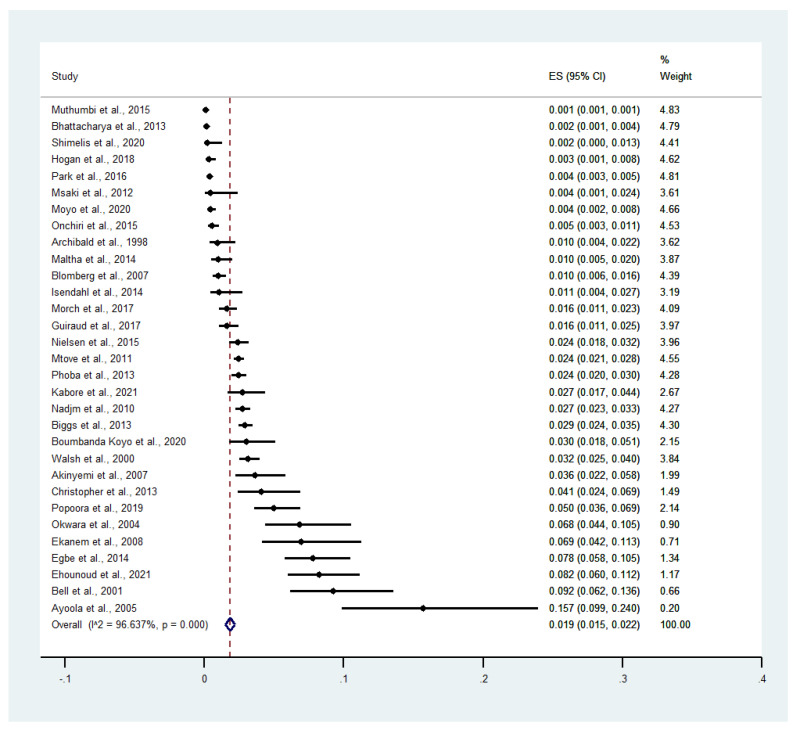
The pooled prevalence of malaria and bacteremia co-infection among febrile patients. Abbreviations: ES: Prevalence estimate; CI: confidence interval.

**Figure 4:**
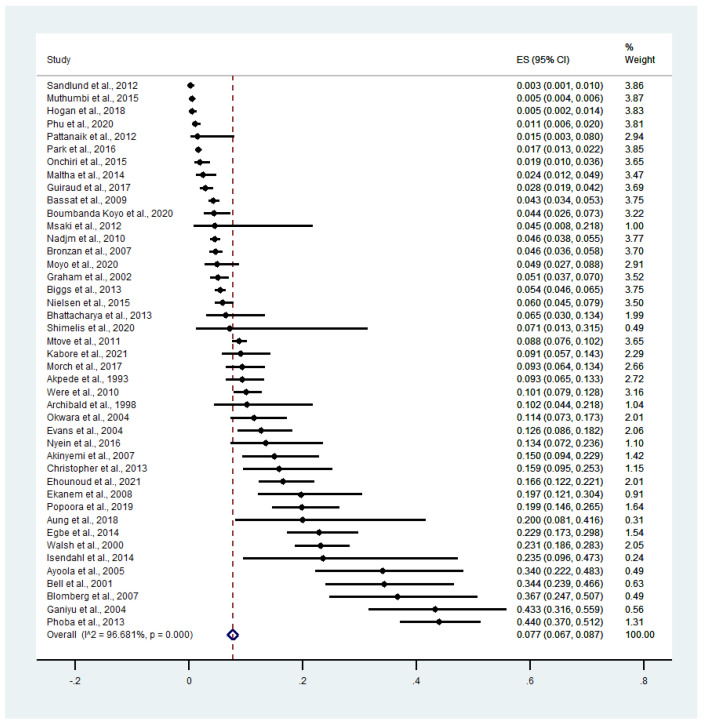
The pooled prevalence of bacteremia among patients with malaria. Abbreviations: ES: Prevalence estimate; CI: confidence interval.

Of the 51 included studies, 31 studies that reported the number of patients co-infected with malaria and bacteremia were included for meta-analysis of the pooled prevalence of malaria and bacteremia among febrile patients. The results illustrated that the pooled prevalence of co-existent malaria and bacteremia among febrile patients was 1.9% (95% confidence interval (CI) = 1.5–2.2%, *I*^2^ = 96.64%, 31 studies, Figure 3). The subgroup of the continent showed that the pooled prevalence of co-existent malaria and bacteremia among febrile patients was 2.0% in Africa (95% CI = 1.7–2.4%, and *I*^2^ = 96.78%, 29 studies) and 0.2% in Asia (95% CI = 1–4%, *I*^2^ = 99.89%, 2 studies) (Supplementary Figure S1). The pooled prevalence of co-existent malaria and bacteremia was 1.4% among adults (95% CI = 0.6–2.2%, *I*^2^ = 99.89%, 2 studies), 2.0% among children (95% CI = 1.4–2.5%, *I*^2^ = 93.6%, 22 studies), and 1.0% in all age groups (95% CI = 0.6–1.4%, *I*^2^ = 96.21%, 7 studies, Figure 4). According to malaria diagnostic methods, the pooled prevalence of co-existent malaria and bacteremia among febrile patients was 1.8% by microscopy (95% CI = 1.4–2.2%, and *I*^2^ = 94.58%, 19 studies), 1.8% by microscopy/RDT (95% CI = 0.8–2.7%, *I*^2^ = 97.93%, 7 studies), 3.7% by Polymerase Chain Reaction (PCR) (95% CI = 0–0.5%, *I*^2^ = 95.4%, 3 studies), and 1.8% by microscopy/RDT/PCR (95% CI = 1.2–2.4%, *I*^2^ = 97.7%, 2 studies, Supplementary Figure S2). For bacteremia diagnostic methods, the pooled prevalence of co-existent malaria and bacteremia among febrile patients was 1.6% by blood culture (95% CI = 1.3–1.9%, *I*^2^ = 96.72%, 27 studies), 4.5% by PCR (95% CI = 3.1–5.9%, *I*^2^ = 99.87%, 2 studies), and 3.6% by blood culture/PCR (95% CI = 2.6–4.6%, *I*^2^ = 99.87%, 2 studies, Supplementary Figure S3).

### 3.6. Prevalence of Bacteremia among Patients with Malaria

The prevalence of bacteremia among patients with malaria included 43 studies; these studies investigated bacteremia among patients with malaria. The results illustrated that the pooled prevalence of bacteremia among patients with malaria was 7.6% (95% CI = 6.7–8.7%, *I*^2^ = 96.68%, 43 studies, Figure 4). The subgroup of the continent showed that the pooled prevalence of co-existent malaria and bacteremia among febrile patients was 9.1% in Africa (95% CI = 7.8–10.4%, *I*^2^ = 97.11%, 37 studies) and 6.1% in Asia (95% CI = 2.1–10.1%, *I*^2^ = 86.54%, 6 studies, Supplementary Figure S4). The pooled prevalence of bacteremia was 10.3% among adults with malaria (95% CI = 4.4–16.3%, *I*^2^ = 89.70%, 6 studies), 9.1% among children with malaria (95% CI = 7.4–10.8%, *I*^2^ = 95.52%, 27 studies), and 5.7% in all age groups (95% CI = 4.3–7.1%, *I*^2^ = 96.06%, 11 studies, Supplementary Figure S5). The pooled prevalence of bacteremia among patients with malaria was 8.3% by microscopy (95% CI = 7.0–9.6%, *I*^2^ = 96.05%, 29 studies), 8.5% by microscopy/RDT (95% CI = 5.2–11.8%, *I*^2^ = 97.24%, 6 studies), 8.2% by PCR (95% CI = 2.3–14.1%, *I*^2^ = 90.01%, 3 studies), and 4.8% by microscopy/RDT/PCR (95% CI = 0.1–9.5%, *I*^2^ = 92.9%, 4 studies, Supplementary Figure S6). The pooled prevalence of bacteremia among patients with malaria was 7.2% by blood culture (95% CI = 6.2–8.3%, *I*^2^ = 96.74%, 39 studies), 6.6% by PCR (95% CI = 4.5–8.7%, *I*^2^ = 99.91%, 2 studies), and 12.8% by blood culture/PCR (95% CI = 9.3–16.2%, *I*^2^ = 99.91%, 2 studies, Supplementary Figure S7). The pooled prevalence of bacteremia among patients with severe malaria was 3.7% (95% CI =1.6–5.8%, *I*^2^ = 94.19%, 4 studies, Supplementary Figure S8). The pooled prevalence of bacteremia was 5.0% among children with severe malaria (95% CI = 4.0–5.0%, *I*^2^ = 0%, 3 studies) and 1.0% among children with severe malaria (95% CI = 1.0–2.0%, Supplementary Figure S9).

### 3.7. Odds of Malaria and Bacteremia Co-Infections

There was significant odds of co-infection (*p* = 0.024, odds ratio (OR) = 0.64, 95% CI = 0.43–0.94, and *I*^2^ = 95.7%, 29 studies, Supplementary Figure S10). Significant odds of co-infection were found in Asia (*p* = 0.54, OR = 1.13, 95% CI = 0.76–1.70, and *I*^2^ = 0%, 2 studies) but not found in Africa (*p* = 0.019, OR = 0.61, 95% CI = 0.41–0.92, and *I*^2^ = 95.9%, 27 studies, Supplementary Figure S11). The significant odds of co-infection was found in in adults (*p* = 0.877, OR = 0.84, 95% CI = 0.09–7.59, and *I*^2^ = 93.2%, 2 studies) and in all age groups (*p* = 0.366, OR = 0.66, 95% CI = 0.27–1.63, and *I*^2^ = 97.3%, 7 studies) but not found in children (*p* = 0.035, OR = 0.61, 95% CI = 0.38–0.97, and *I*^2^ = 95.0%, 20 studies, Supplementary Figure S12).

### 3.8. Clinical Outcomes of Co-Infection

Fourteen studies [26,34,35,37,39,41,42,55,61,62,69,74,75,76] reported the clinical outcomes of patients with co-infection. Most studies (9/14, 64.3%) [35,39,41,42,55,61,62,69,76] reported a higher proportion of deaths among co-infected patients. Meanwhile, the remaining studies (5/14, 35.7%) reported clinically severe disease and high rates of malaria parasitemia [26], diarrhea and restlessness [34], *P*. *vivax* relapse [37], splenomegaly and anemia [75], and persistent malaria-like symptoms after treatment [74]. The pooled proportion of deaths was estimated using the available data on patients who died because of co-infection in 8 studies [26,35,41,42,61,62,69,76]. The results demonstrated that the pooled proportion of deaths among patients with co-infection was 15.0% (95% CI = 8.0–23.0%, *I*^2^ = 75.23%, 8 studies, Supplementary Figure S13).

### 3.9. Publication Bias

The publication bias of the probability of co-existent malaria and bacteremia among febrile patients was assessed using the funnel plot and Egger’s test. The results revealed the symmetrical distribution of the effect estimates (estimated OR) from the middle line (Supplementary Figure S14). Egger’s test demonstrated no significant small-study effect (*p* = 0.184), indicating the absence of publication bias.

## 4. Discussion

The present study demonstrated that most patients with malaria, particularly those infected by *P*. *falciparum*, were co-infected by Gram-negative bacteria, typically typhoidal *Salmonella*, whereas among patients with *P*. *falciparum* and Gram-positive bacteria co-infection were primarily infected by *S. aureus*. This result was in accordance with that of a previous systematic review demonstrating that malaria was foundlikely to co-exist with both of Gram-positive bacteria and Gram-negative bacteria [25]. Malaria co-existing with Gram-negative bacteria such as NTS, *E*. *coli*, and *K*. *pneumoniae* or Gram-positive bacteria such as *S*. *pneumoniae* and *Staphylococcus* spp. has also been reported, albeit at lower proportions. Typhoidal *Salmonella* and NTS are the most common pathogens isolated from the blood of patients [35,41,76].

In the meta-analysis approach, the present results indicated that co-existent malaria and bacteremia are uncommon among febrile patients presenting to healthcare facilities, but co-infection was mainly reported among febrile patients in Africa. In addition, co-infection was more common in children than in adults. This result was in accordance with previous findings demonstrating that bacteria, particularly NTS, were most frequently isolated among children younger than 5 years with malaria [20,25,66]. Interestingly, the prevalence of co-infection was higher in studies using PCR as a diagnostic tool for malaria or bacteremia than in studies using other methods such as microscopy. This result indicated that molecular techniques should be applied to detect malaria or bacteremia in endemic areas. Although blood culture is the gold standard for detecting bacteremia, it does not permit the isolation of intracellular or highly fastidious bacteria. Therefore, molecular strategies such as PCR could help identify fastidious bacteria such as *Rickettsia felis* and *Tropheryma whipplei*, as reported in previous studies [57,77], and increase the detection sensitivity versus conventional methods. Nevertheless, blood culture is extremely valuable in areas of limited resources, in which the causes of febrile illness with concomitant parasitemia are difficult to identify [49,78]. This was the probable reason why the prevalence of co-infection reported by studies using PCR was higher than that reported by studies using blood culture, which only detects common bacteria such as *Salmonella*, *S. pneumoniae*, or *S. aureus* [79,80]. The low prevalence of concomitant malaria and bacteremia in some studies included in the meta-analysis might have contributed to the frequent use of antibiotics among patients before hospitalization for bacteremia because some bacteria such as pneumococci are susceptible to antibiotics [26]. The reduction in the prevalence of malaria by national malaria control strategies also influenced the low prevalence of co-infection in African countries [59,81]. Another reason for the difference in the prevalence of co-infection among studies might be the difference in the size of the hospitals to which patients were admitted as severely ill patients were more likely to be admitted to tertiary hospitals than to regional hospitals. Because of this, higher reports of patient mortality were documented in tertiary hospitals compared to regional hospitals [26].

The present study recorded a low prevalence of bacteremia among patients with malaria, and co-infection was mainly reported among febrile patients in Africa and Asia. Previous studies demonstrated that malaria was associated with bacterial infection, particularly in areas in which malaria is endemic among children such as Africa [10,25,35,41,55,76,82]. Meanwhile, in Asia, a study of Vietnamese adults with severe falciparum malaria demonstrated a low rate of concomitant bacteremia of approximately 1% [69]. In addition, a study in Myanmar reported that approximately 20% of patients with severe malaria had bacteremia [33]. The difference in prevalence between these two studies might be explained by the difference in the strictness of the criteria for severe malaria. The study in Vietnamese adults used stricter criteria for severe malaria; hence, a lower prevalence of severe malaria was reported [69]. In addition, there is a difference between malaria and bacterial infections between rural [35,38,40,41,44,48,49,50,52,54,55,56,57,59,60,61,62,65,66,68,76] and urban areas [37,38,41,44,45,57,66,72]. The higher prevalence was found in rural areas than in urban areas. The increasing number of cases of malaria and co-infection is associated with factors including improper sewage disposal, poor personal hygiene, and poverty particular in rural areas [29]. Our results also demonstrated a higher prevalence of bacteremia among patients with malaria in studies using microscopy/RDT/PCR as diagnostic tools for malaria or bacteremia. Therefore, this finding supported the use of molecular techniques such as quantitative PCR for identifying co-infection, helping to differentiate severe from non-severe malaria [83], and further guiding the management of malaria and bacteremia co-infection in febrile patients.

The present study demonstrated that the pooled prevalence of bacteremia in patients with severe malaria was higher in children than in adults. Previous studies suggested that bacteremia among patients with malaria was related to decreasing parasite densities [49,52]. The decreased in parasite densities among children with severe concomitant febrile infection might be due to non-malarial diseases might reducing the likelihood of malaria infection and/or severity, or selection bias in the study [49]. However, another study found that bacteremia in patients with malaria can lead to clinically severe disease and severe malaria parasitemia [26]. In another manner, severe parasitemia in patients with malaria can lead to an increased risk of bacteremia because of the more intense parasitized sequestration in the gastrointestinal tract or other organs [84]. In addition, the risk of bacteremia was increased in patients with severe malaria, who exhibit immune-mediated barrier dysfunction in the gastrointestinal tract [85,86]. Therefore, antibiotic treatment may have been needed in all children and some adults with severe malaria who experience deterioration or extremely severe malaria parasitemia [84]. Regarding the difference in antibiotic treatment between children and adults, children who develop severe malaria should receive broad-spectrum antibiotics because the possibility of bacteremia could not be eliminated [12,62]. However, bacteremia in adults with severe malaria has rarely been reported in low-transmission areas such as Asia [67,70]. Therefore, the administration of empiric antibiotics to adults with severe malaria was not recommended unless bacteremia was clearly detected [87].

The present study demonstrated that there were significant odds of co-existent malaria and bacteremia in febrile patients among studies conducted in African children. The increased odds of co-existent malaria and bacteremia was demonstrated in Malawi [36,75], Nigeria [29], and Tanzania [39,42]. Of those studies, the highest odds of co-existent malaria and bacteremia was demonstrated in the prospective cohort study conducted in Tanzania [39]. Conversely, decreased odds of co-existent malaria and bacteremia were demonstrated in Tanzania [26,31,38,59,61], Nigeria [45], Burkina Faso [48,55], Kenya [60], Ghana [49,62], and the Republic of Côte d’Ivoire [44]. Potential reasons for the increased odds of co-existent malaria and bacteremia include that the diseases share similar geographical areas, malaria facilitates bacterial infection, and bacterial infection facilitates malaria. This co-infection should be further investigated by experimental studies.

The present study found that that co-existent malaria and bacteremia resulted in higher case-fatality rates. From the results of the meta-analysis based on a limited number of studies, the highest prevalence of death was demonstrated in a study conducted in Vietnam (44%) [69]. The study reported a low prevalence of bacteremia among patients with severe falciparum malaria; however, among patients with co-infection, case-fatality rates were high, and the risk ratio for death in patients with concomitant bacteremia and severe malaria was 3.44 [69]. The lowest proportion of deaths was reported in Kenya (3%) [76] and Tanzania (8%) [42]. The heterogeneity of the pooled proportion of deaths in the meta-analysis might be explained by the early use of antibiotics in patients with severe malaria in a study without a strict definition of severe falciparum malaria or mild malaria parasitemia [33]. In addition, the study of Vietnamese adults also did not provide antibiotics [69]. Co-existent malaria and bacteremia was reported as a risk factor for poor prognosis [25,59]. Nevertheless, the low proportion of deaths in some studies might have contributed to a general reduction in the incidence of malaria because of effective malaria control programs. The high proportion of deaths among co-infected patients was caused by late presentation to the hospital, which was an independent risk factor for death in one study [26]. Conversely, a proportion of deaths was noted in clinical trials in which patients received closer attention than provided in other study designs [33].

The present study had several limitations. First, the searches in gray literature such as Google scholar could not be performed. The keywords such as (malaria) AND (leptospirosis), (malaria) AND (*Brucellosis*) were not be used in our searches; therefore, they might be used in further systematic reviews to retrieve more relevant studies. Second, data were pooled from studies with different study sites, methodologies, and participants, and thus extreme caution must be exercised in interpreting the findings. Third, the clinical outcomes among patients with co-infection were understudied; hence, the outcomes of co-infected patients included only the number of deaths, which can be caused by several confounding factors. Fourth, the pooled prevalence of malaria and bacteremia co-infections among febrile patients in Asia was based on only two studies and thus is not generalizable to the whole continent.

## 5. Conclusions

On the basis of this study, we conclude that although the prevalence of co-infection was low, patients with severe malaria have a higher risk of death because of co-existent bacteremia. As the prevalence of deaths among patients with co-infections was high (15%), antibiotics for bacteremia prophylaxis may be more useful in both children and adults with severe malaria.

## Figures and Tables

**Table 1:** Characteristics of malaria and bacteremia co-infection cases.

Characteristics of Malaria and Bacteremia	N (%)
*P. falciparum* and Gram-negative bacteria	648
- Typhoidal *Salmonella*	414
- *Salmonella* spp.	74
- Non-typhoidal *Salmonella*	19
- *Escherichia coli*	10
- *Klebsiella pneumoniae*	12
- *Rickettsia felis*	12
- *Acinetobacter* spp.	6
- *Klebsiella* spp.	4
- *Haemophilus influenzae*	3
- *Pseudomonas* spp.	3
- *Enterobacter cloacae*	2
- *Serratia marcescens*	2
- *Aeromonas* spp.	1
- *Campylobacter* spp.	1
- *Providencia* spp.	1
- *Alcaligenes faecalis*	1
- *Ochrobactrum tritici*	1
- *Shigella* spp.	1
- *Chryseobacter* spp.	1
- *Citrobacter koseri*	1
- Unidentified	79
*P. falciparum* and bacteremia (unidentified bacteria)	391
*P. falciparum* and Gram-positive bacteria	176
- *Staphylococcus aureus*	79
- *Streptococcus pneumoniae*	44
- *Streptococcus* spp.	11
- *Enterococcus* spp.	2
- Unidentified	40
Malaria (unidentified species) and Gram-negative bacteria	152
- Typhoidal *Salmonella*	47
- Non-typhoidal *Salmonella*	41
- *Salmonella* spp.	1
- *E. coli*	11
- *K. pneumoniae*	10
- *Klebsiella* spp.	1
- *Proteus* spp.	4
- *E. cloacae*	2
- *Acinetobacter* spp.	2
- *P. aeruginosa*	2
- *H. influenzae*	1
- *Campylobacter* spp.	1
- Unidentified	29
Malaria (unidentified species) and Gram-positive bacteria	51
- *S. aureus*	4
- *S. pneumoniae*	6
- *Staphylococcus* spp.	16
- *Streptococcus* spp.	2
- *Enterococcus* spp.	1
- Unidentified	22
*P. falciparum* and mixed infection	16
- *P. malariae + H. influenzae*	1
- *P. malariae + S. pneumoniae*	1
- *P. ovale + R. felis*	1
- *P. ovale + S. aureus*	2
- *Acinetobacter baumannii, E. cloacae*	1
- *H. influenzae, S. aureus,* and *S. pneumoniae*	1
- *R. felis + S. aureus*	1
- *S. aureus + S. pneumoniae*	5
- *T. whipplei, S. aureus,* and *S. pneumoniae*	1
- *S. aureus and S. pyogenes*	1
- *Escherichia vulneris/Proteus mirabilis*	1
Non-*P. falciparum* and Gram-negative bacteria	5
- *P. vivax +* Typhoidal *Salmonella*	2
- *P. vivax +* other Gram negative bacteria	3
Non-*P. falciparum* and Gram-positive bacteria	1
- *P. vivax +* Gram-positive bacteria	1
Non-*P. falciparum* and mixed infection	1
- *P. ovale + S. aureus + S. enterica* Typhi	1
Malaria (unidentified species) and bacteremia (unidentified bacteria)	142
Total	1583

## Data Availability

All data relating to the present study are available in this manuscript and supplementary files.

## Supplementary Materials

The following supporting information can be downloaded at: https://www.mdpi.com/article/10.3390/tropicalmed7090243/s1, Supplementary Figure S1: A subgroup analysis of the pooled prevalence of malaria and bacteremia co-infection by continents; Supplementary Figure S2: A subgroup analysis of the pooled prevalence of malaria and bacteremia co-infection by malaria diagnostic method; Supplementary Figure S3: A subgroup analysis of the pooled prevalence of malaria and bacteremia co-infection by bacteremia diagnostic method; Supplementary Figure S4: A subgroup analysis of the pooled prevalence of bacteremia among patients with malaria by continents; Supplementary Figure S5: A subgroup analysis of the pooled prevalence of bacteremia among patients with malaria by age groups; Supplementary Figure S6: A subgroup analysis of the pooled prevalence of bacteremia among patients with malaria by malaria diagnostic method; Supplementary Figure S7: A subgroup analysis of the pooled prevalence of bacteremia among patients with malaria by bacteremia diagnostic method; Supplementary Figure S8: The pooled prevalence of bacteremia among patients with severe malaria; Supplementary Figure S9: The pooled prevalence of bacteremia among patients with severe malaria by age groups; Supplementary Figure S10: A probability of co-infection; Supplementary Figure S11: A probability of co-infection by continents; Supplementary Figure S12: A probability of co-infection by age groups; Supplementary Figure S13: The pooled prevalence of deaths among patients with co-infection; Supplementary Figure S14: A funnel plot of the probability of co-infection. Table S1: Search terms; Table S2: Details of the included studies; Table S3: Quality of included studies; PRISMA_2020_abstract_checklist; PRISMA_2020_checklist.
