# Supplementary material for: The Prevalence of Malaria and Bacteremia Co-Infections among Febrile Patients: A Systematic Review and Meta-Analysis
Source: Trop Med Infect Dis. 2022 Sep 13;7(9):243. doi: 10.3390/tropicalmed7090243 (PMC9503679; doi:10.3390/tropicalmed7090243)
Supplement: Supplementary file 1 [file tropicalmed-07-00243-s001.zip › Supplementary figures.pdf]

Supplementary Figures

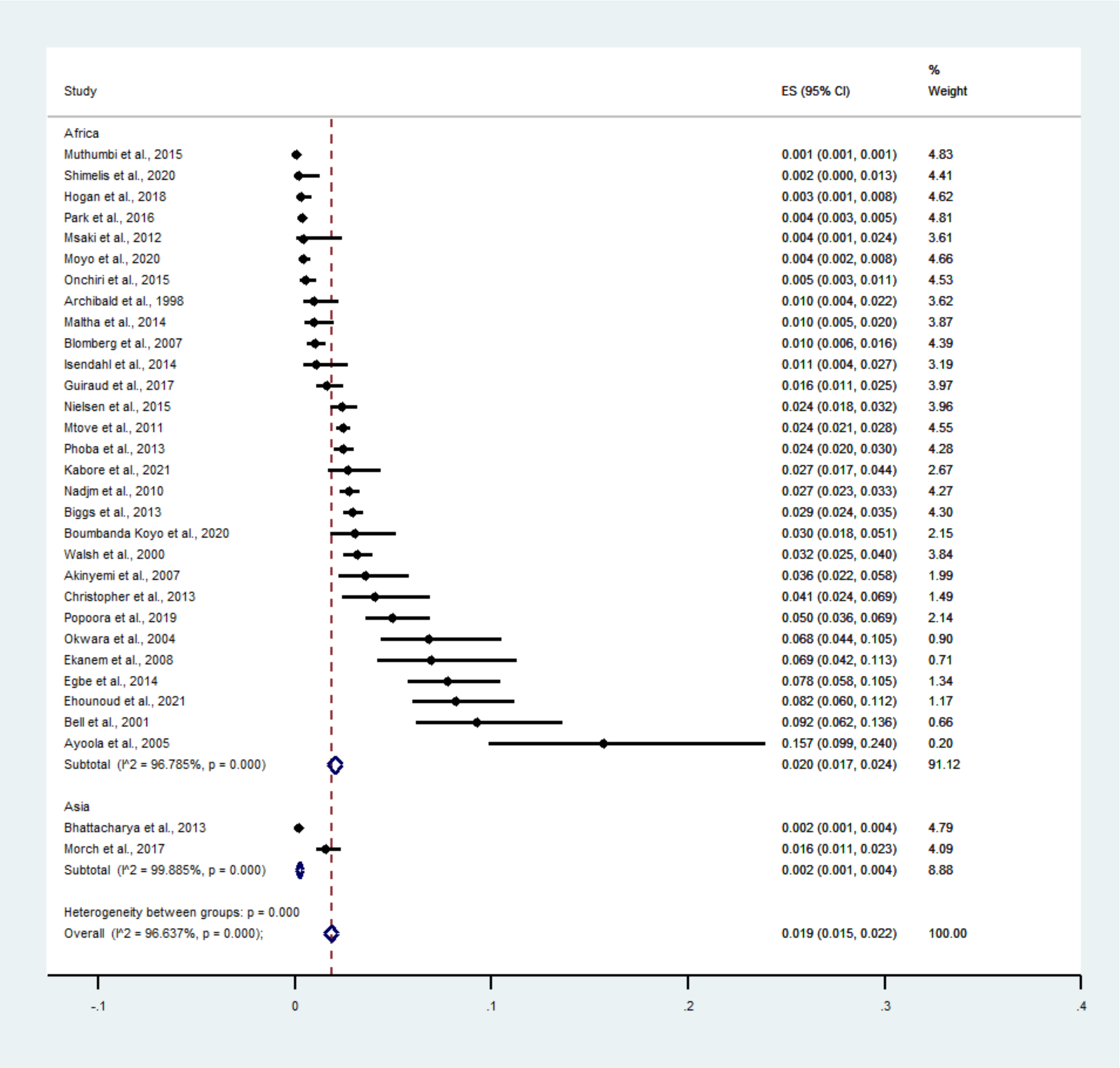

Supplementary Figure S1. A subgroup analysis of the pooled prevalence of malaria and bacteremia co-infection by continents. Abbreviations: ES: prevalence estimate; CI, confidence interval

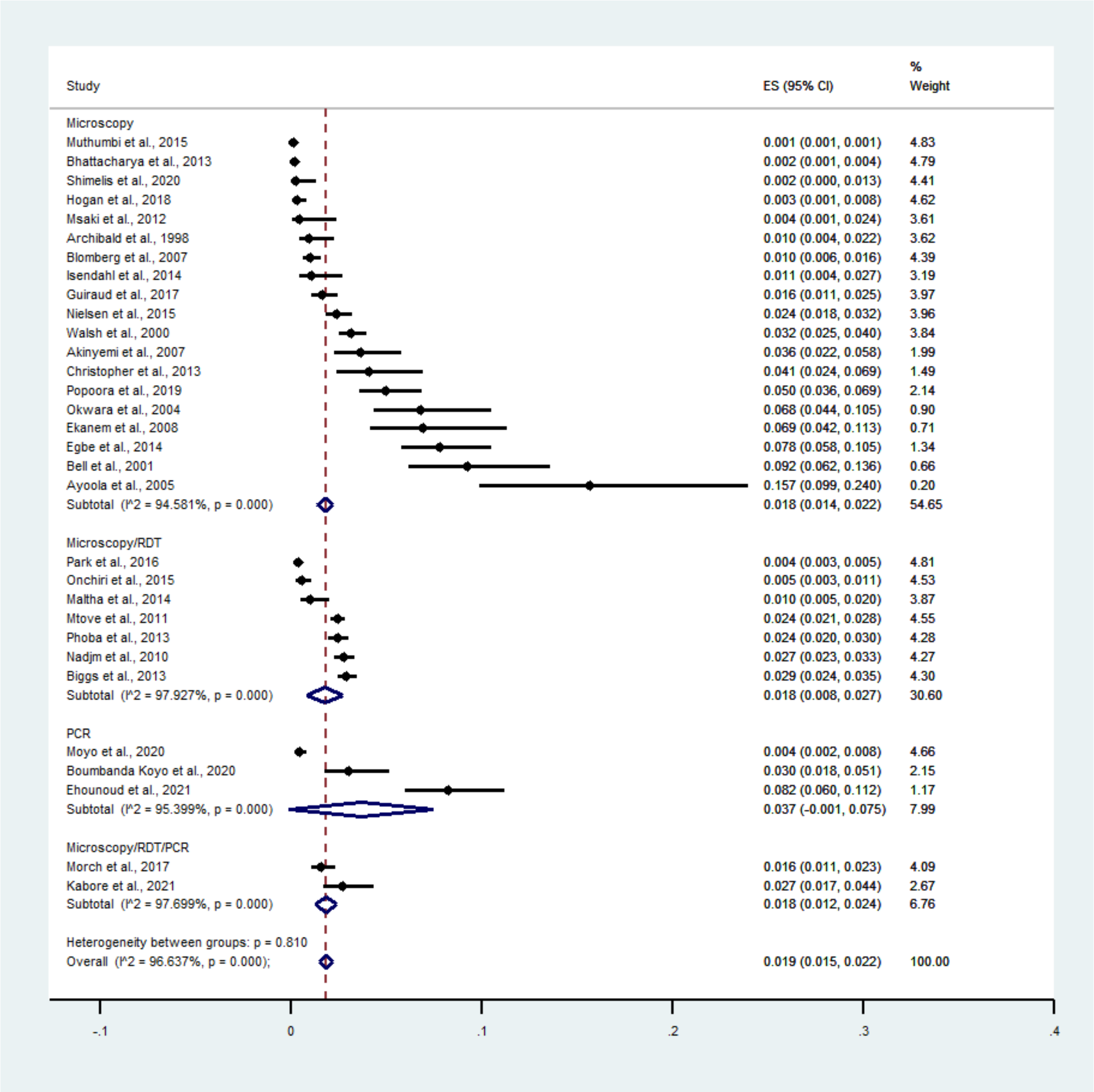

Supplementary Figure S2. A subgroup analysis of the pooled prevalence of malaria and bacteremia co-infection by malaria diagnostic method. Abbreviations: ES: prevalence estimate; CI, confidence interval

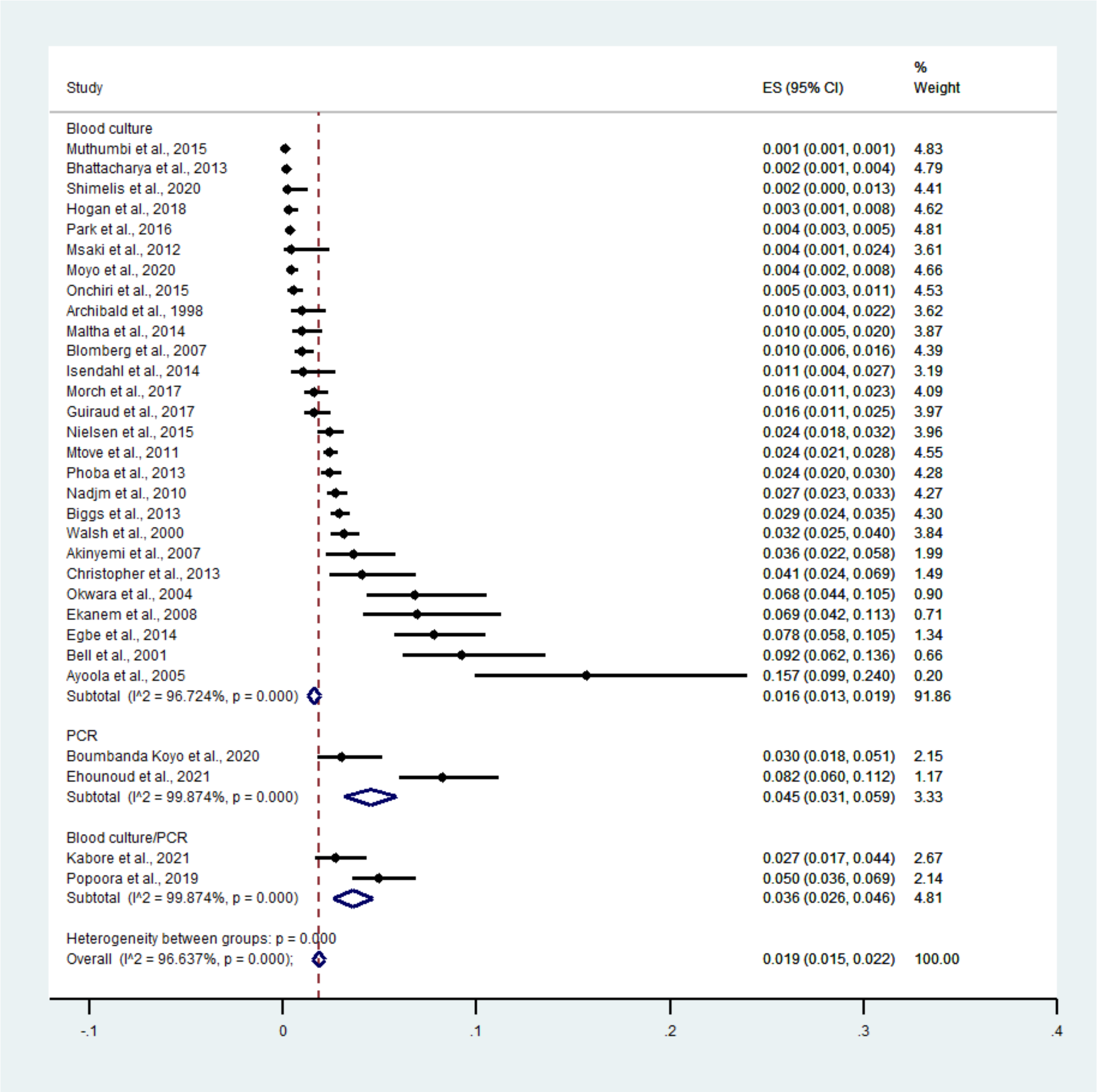

Supplementary Figure S3. A subgroup analysis of the pooled prevalence of malaria and bacteremia co-infection by bacteremia diagnostic method. Abbreviations: ES: prevalence estimate; CI, confidence interval

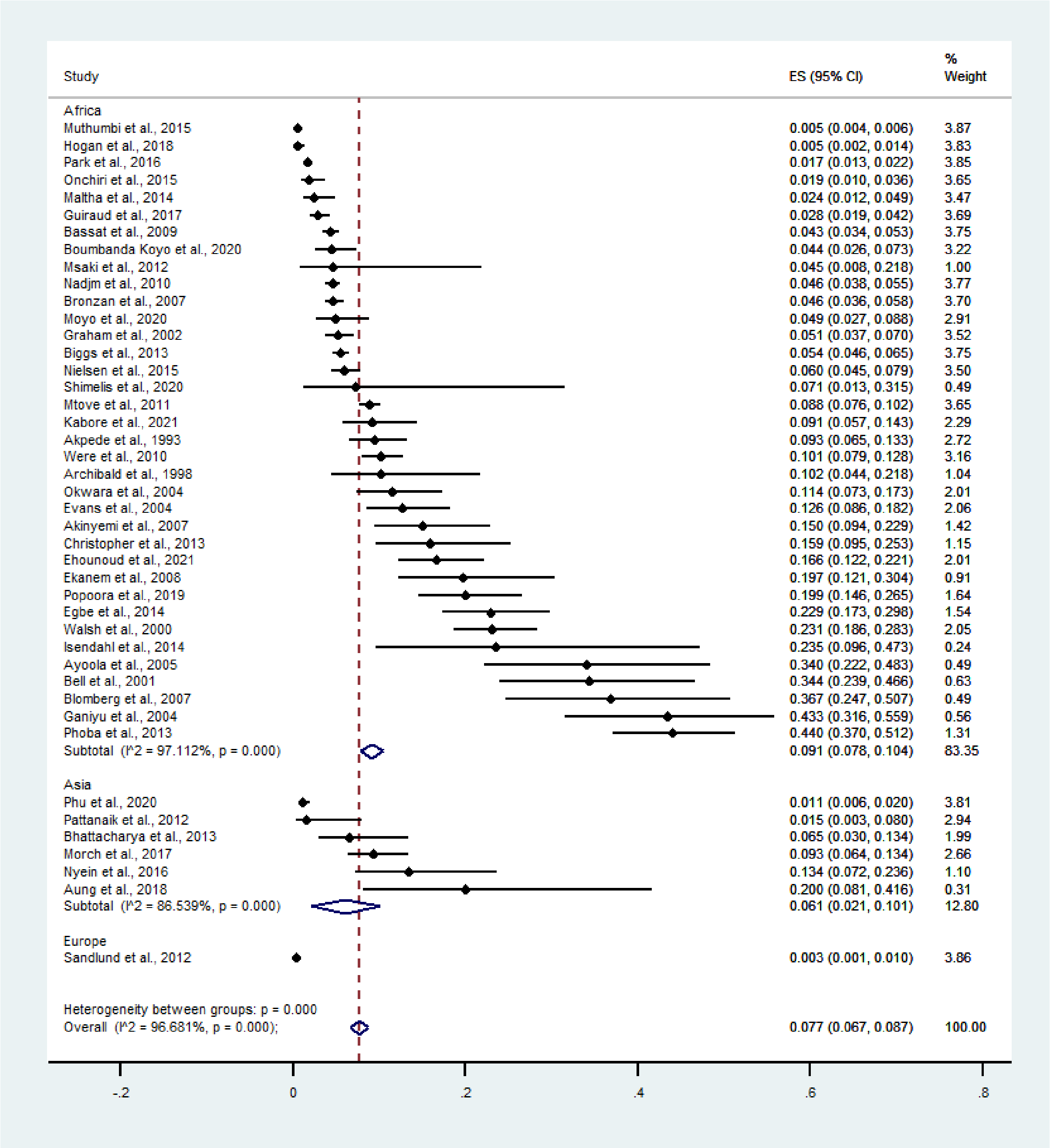

Supplementary Figure S4. A subgroup analysis of the pooled prevalence of bacteremia among patients with malaria by continents. Abbreviations: ES: prevalence estimate; CI, confidence interval

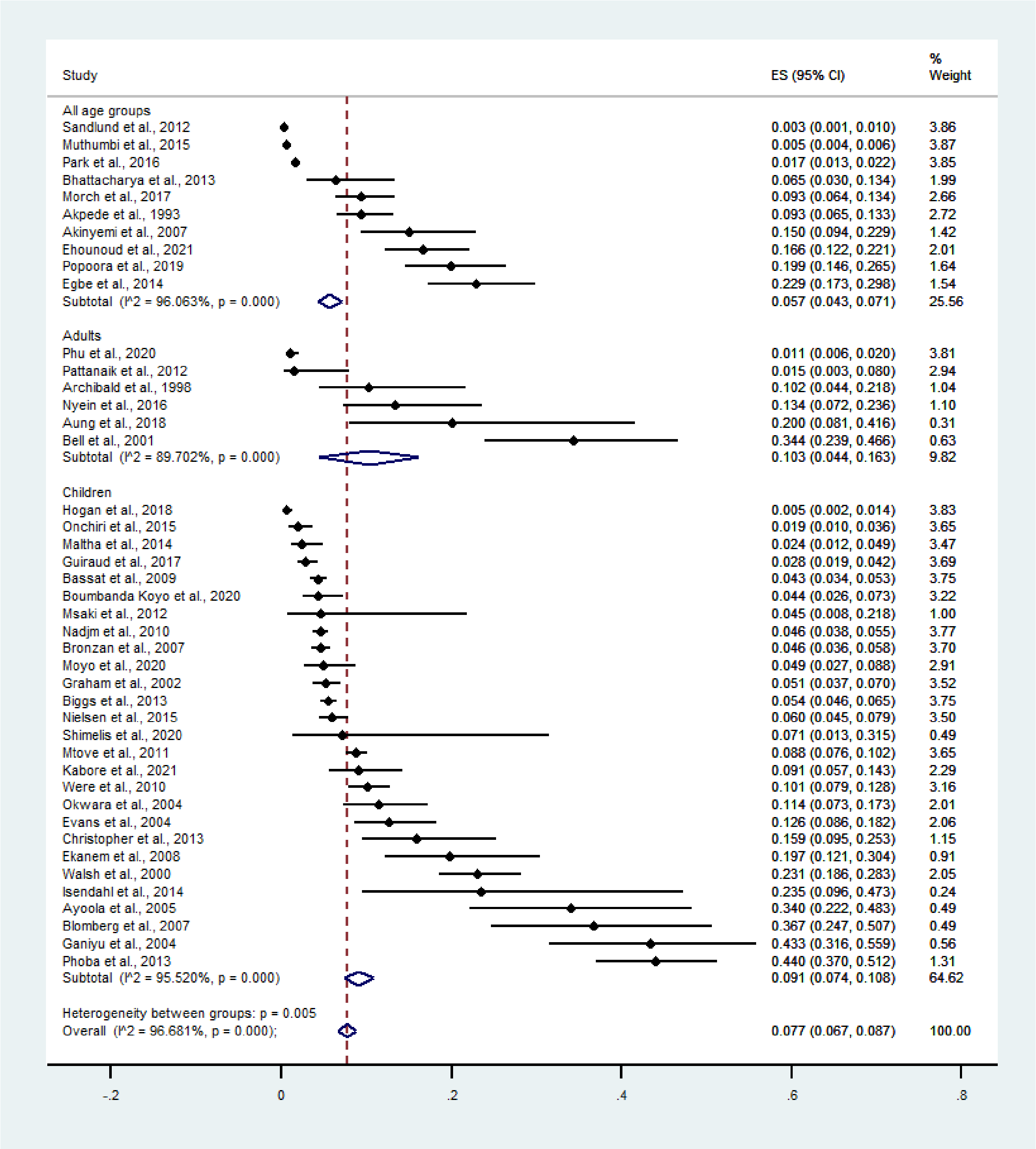

Supplementary Figure S5. A subgroup analysis of the pooled prevalence of bacteremia among patients with malaria by age groups. Abbreviations: ES: prevalence estimate; CI, confidence interval

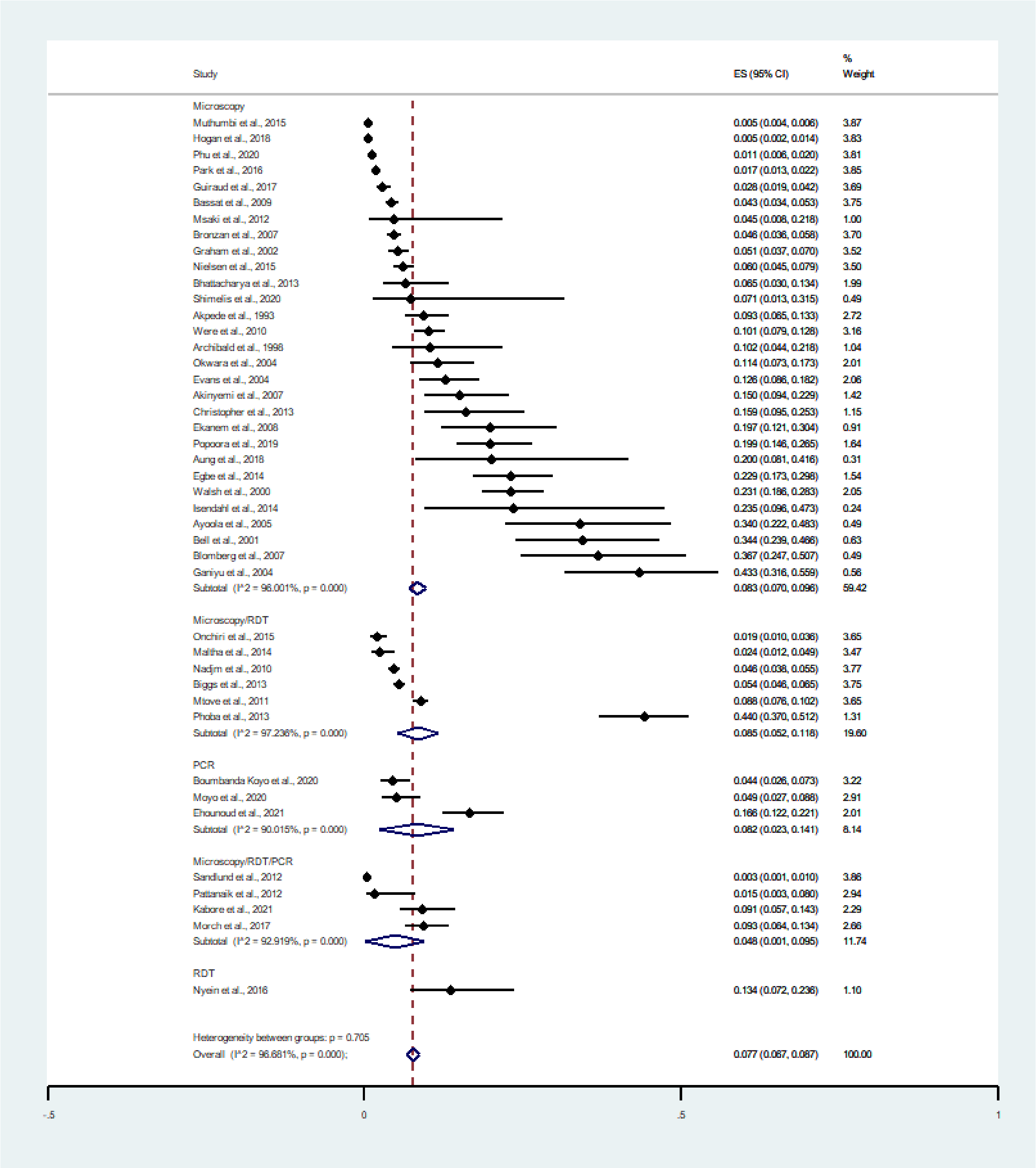

Supplementary Figure S6. A subgroup analysis of the pooled prevalence of bacteremia among patients with malaria by malaria diagnostic method. Abbreviations: ES: prevalence estimate; CI, confidence interval

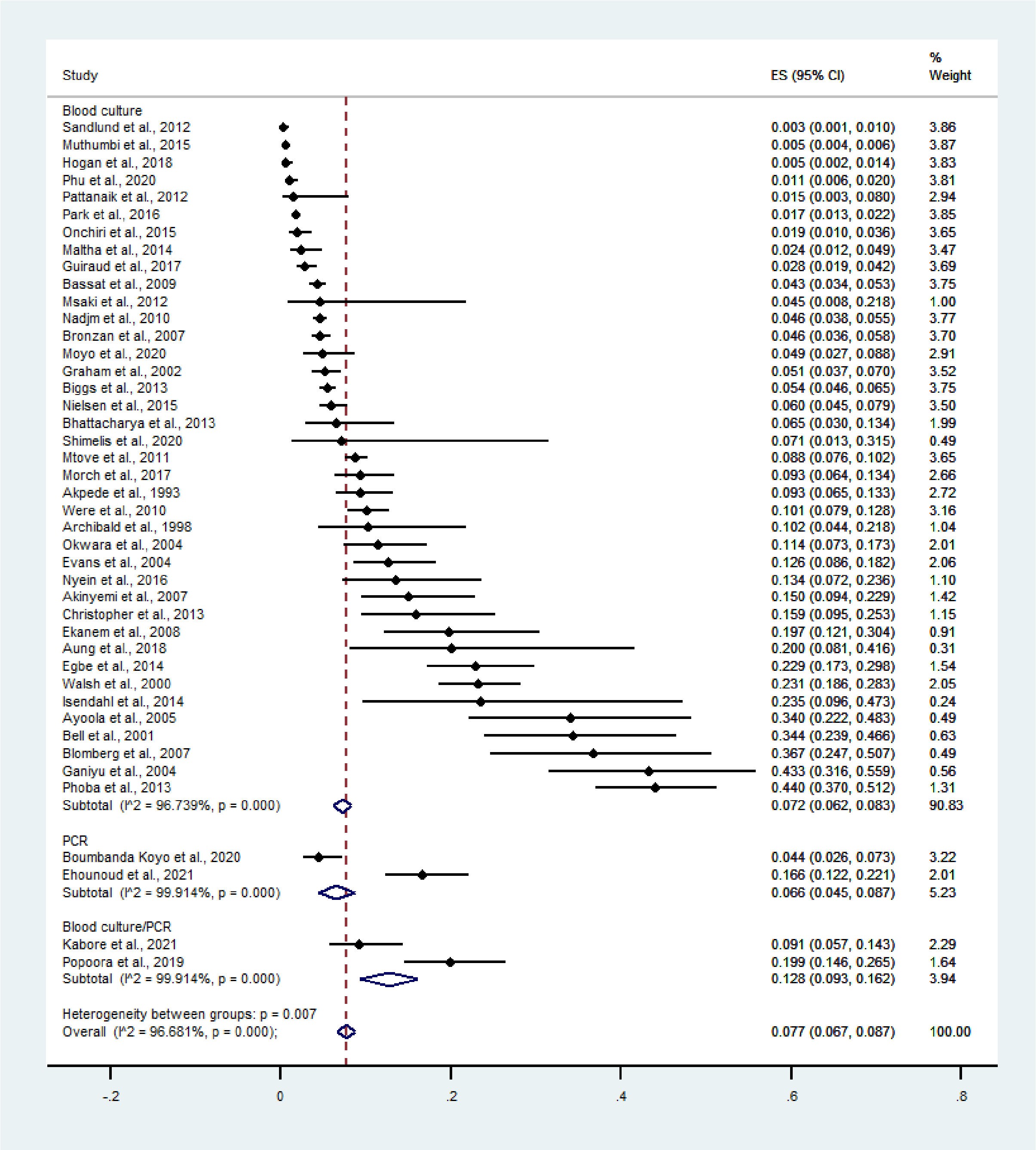

Supplementary Figure S7. A subgroup analysis of the pooled prevalence of bacteremia among patients with malaria by bacteremia diagnostic method. Abbreviations: ES: prevalence estimate; CI, confidence interval

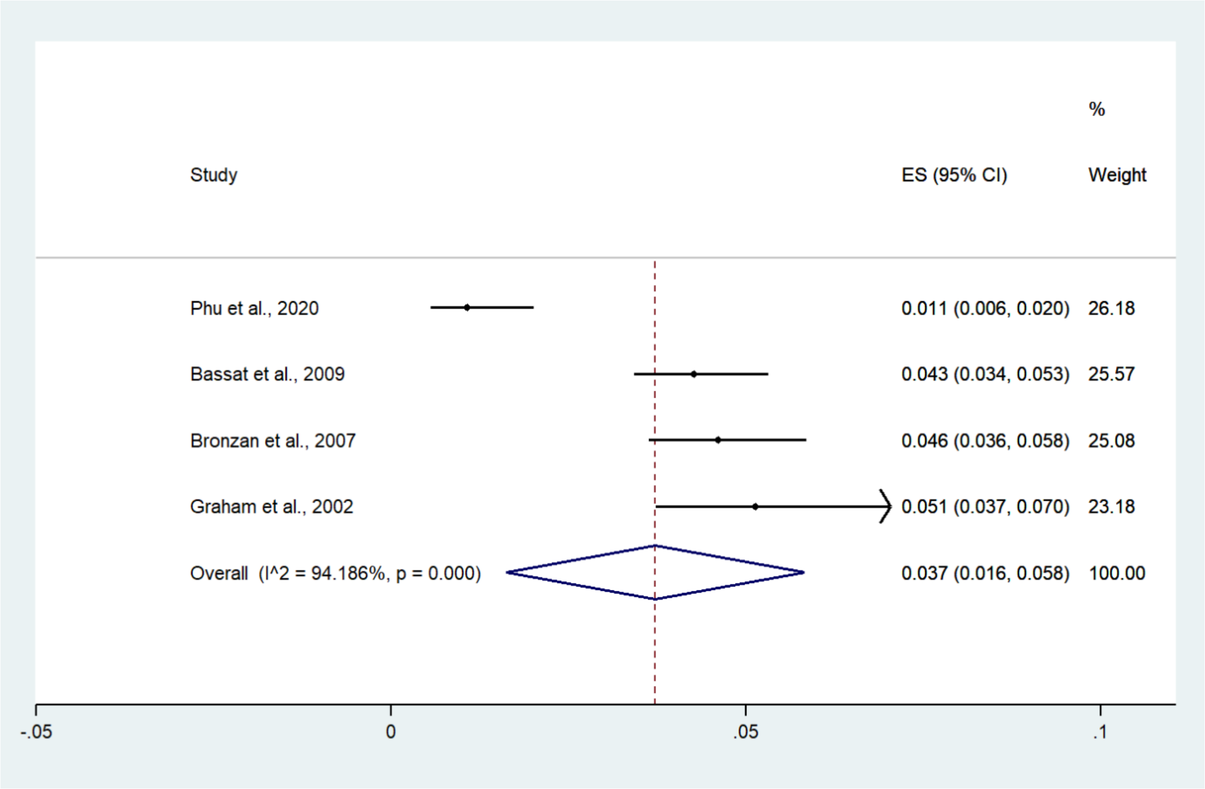

Supplementary Figure S8. The pooled prevalence of bacteremia among patients with severe malaria. Abbreviations: ES: prevalence estimate; CI, confidence interval

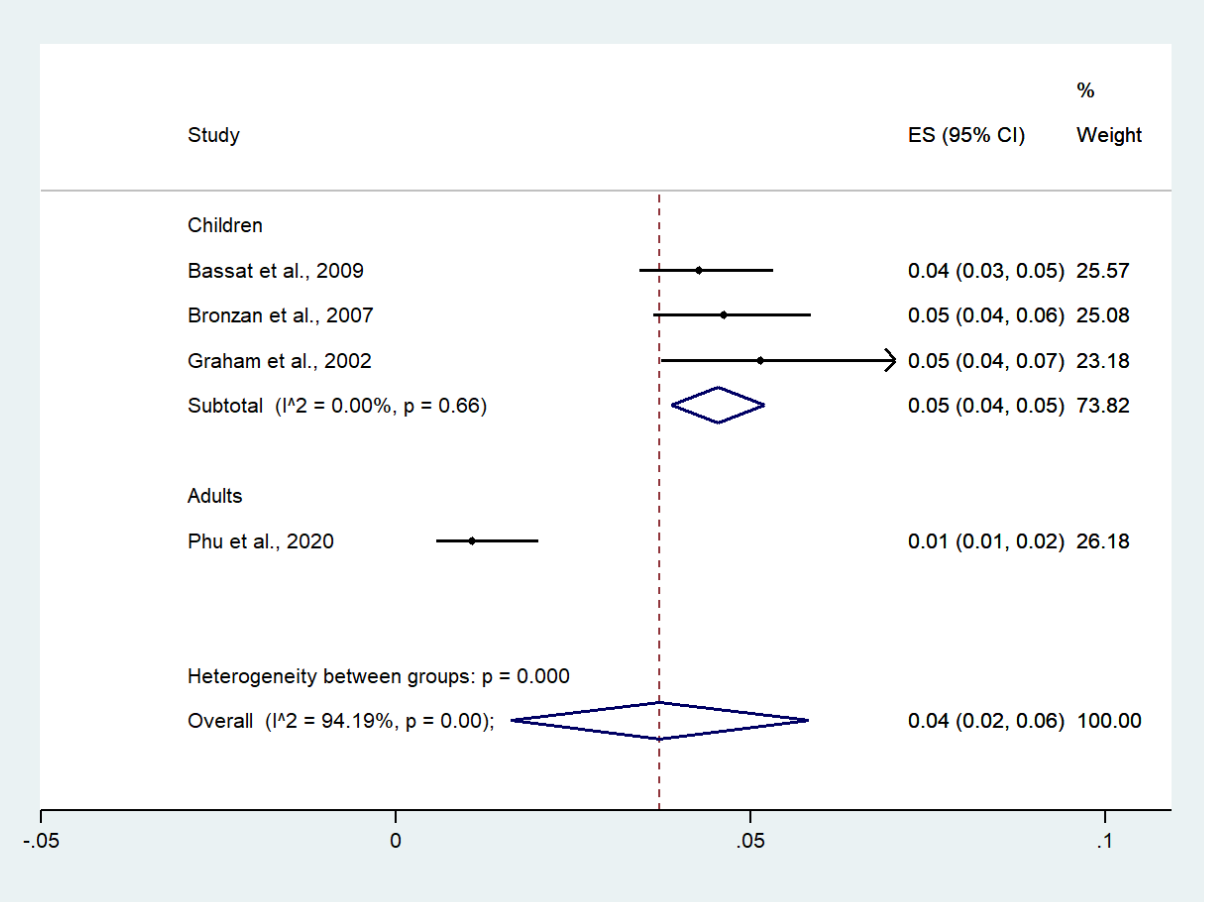

Supplementary Figure S9. The pooled prevalence of bacteremia among patients with severe malaria by age groups. Abbreviations: ES: prevalence estimate; CI, confidence interval

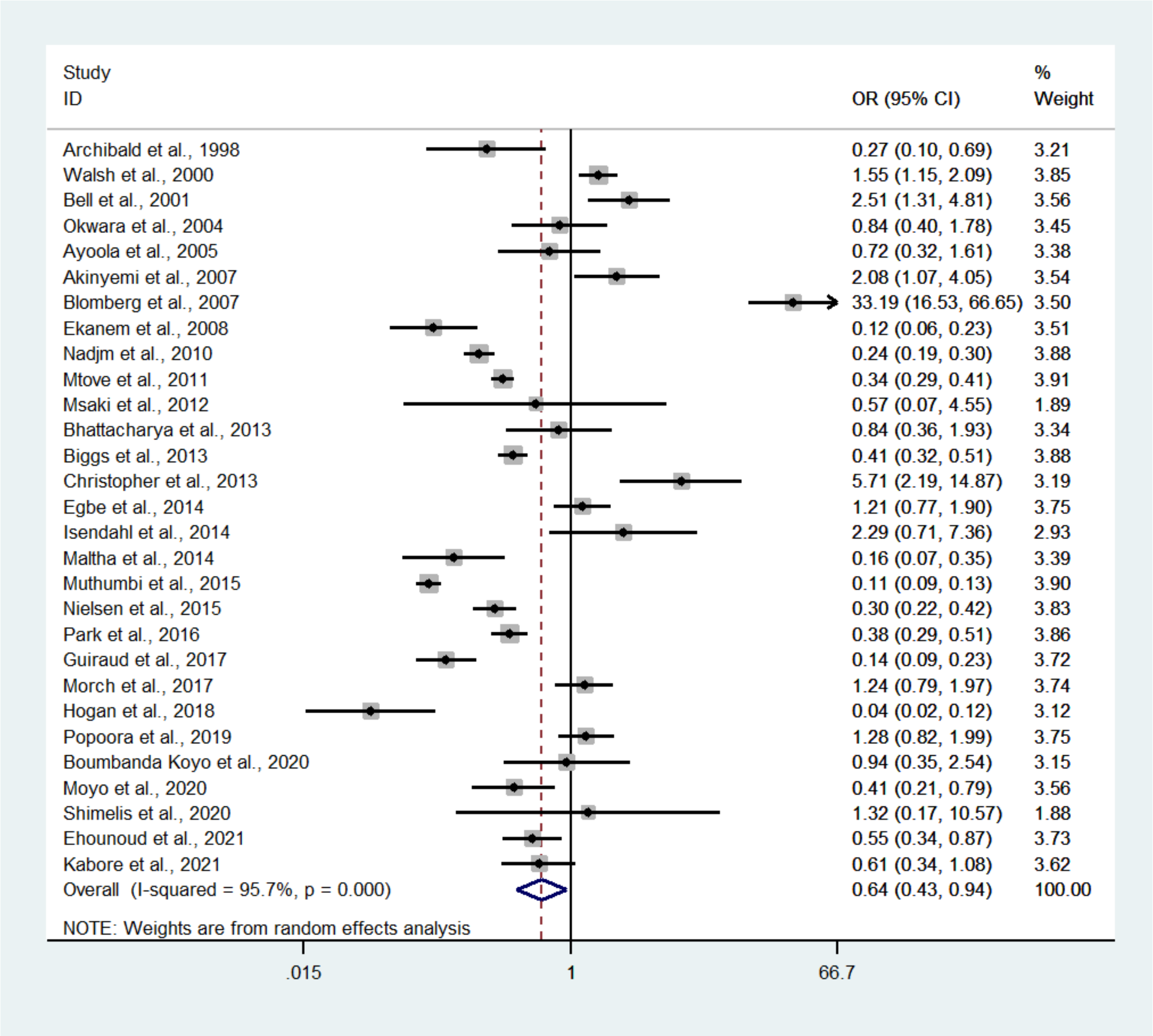

Supplementary Figure S10. A probability of co-infection. Abbreviations: OR: odds ratio; CI, confidence interval

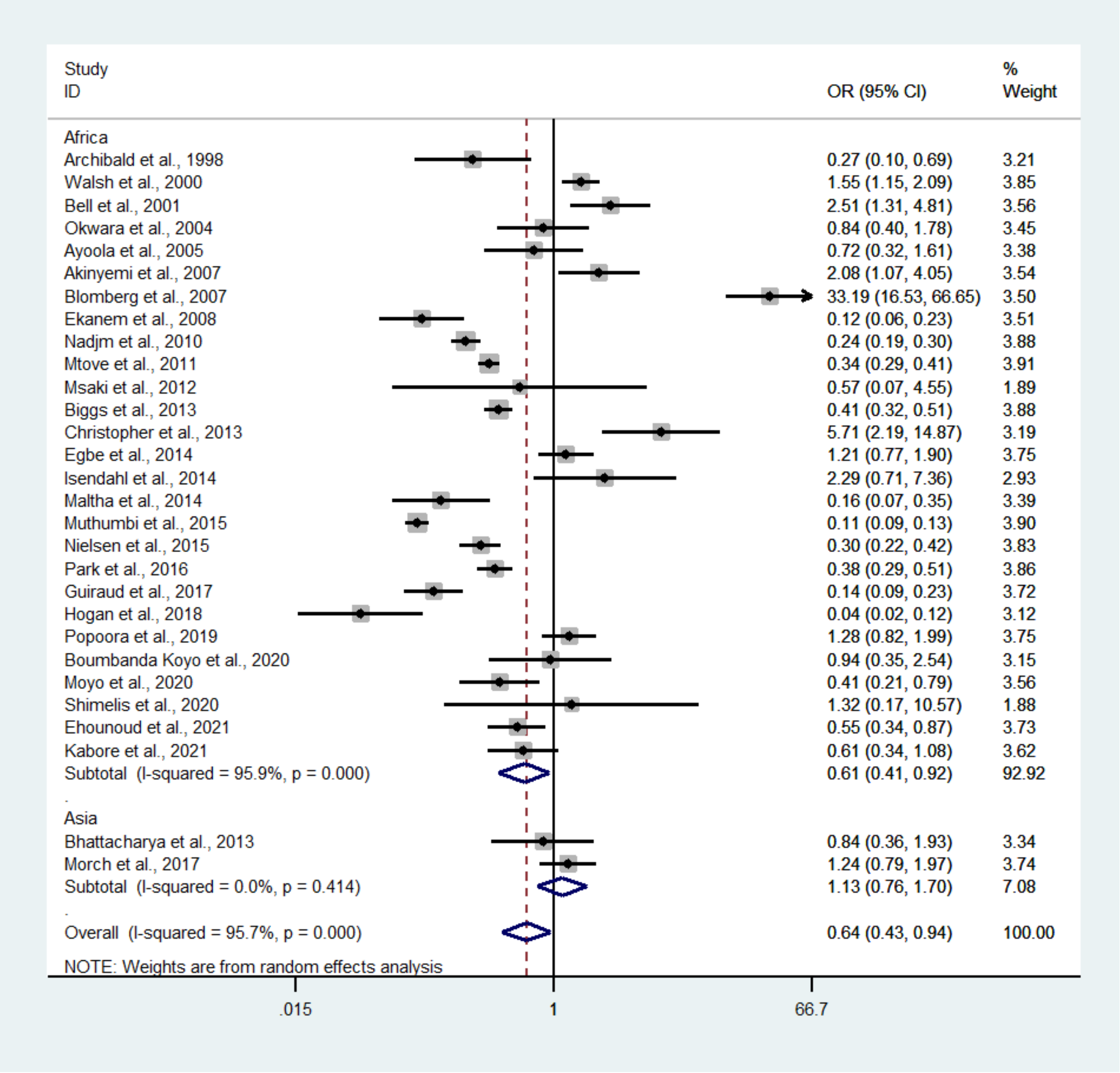

Supplementary Figure S11. A probability of co-infection by continents. Abbreviations: OR: odds ratio; CI, confidence interval

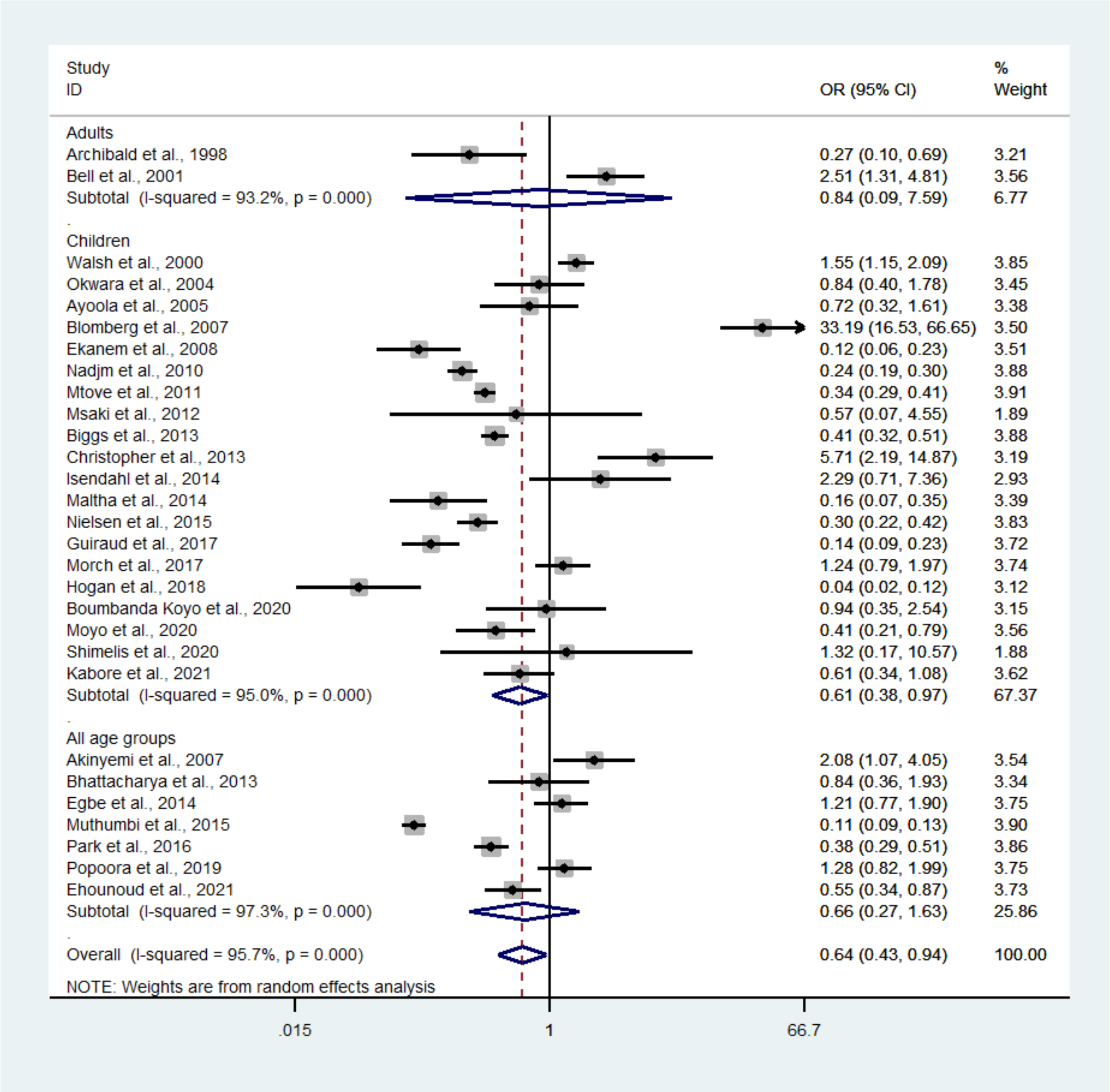

Supplementary Figure S12. A probability of co-infection by age groups**.** Abbreviations: OR: odds ratio; CI, confidence interval

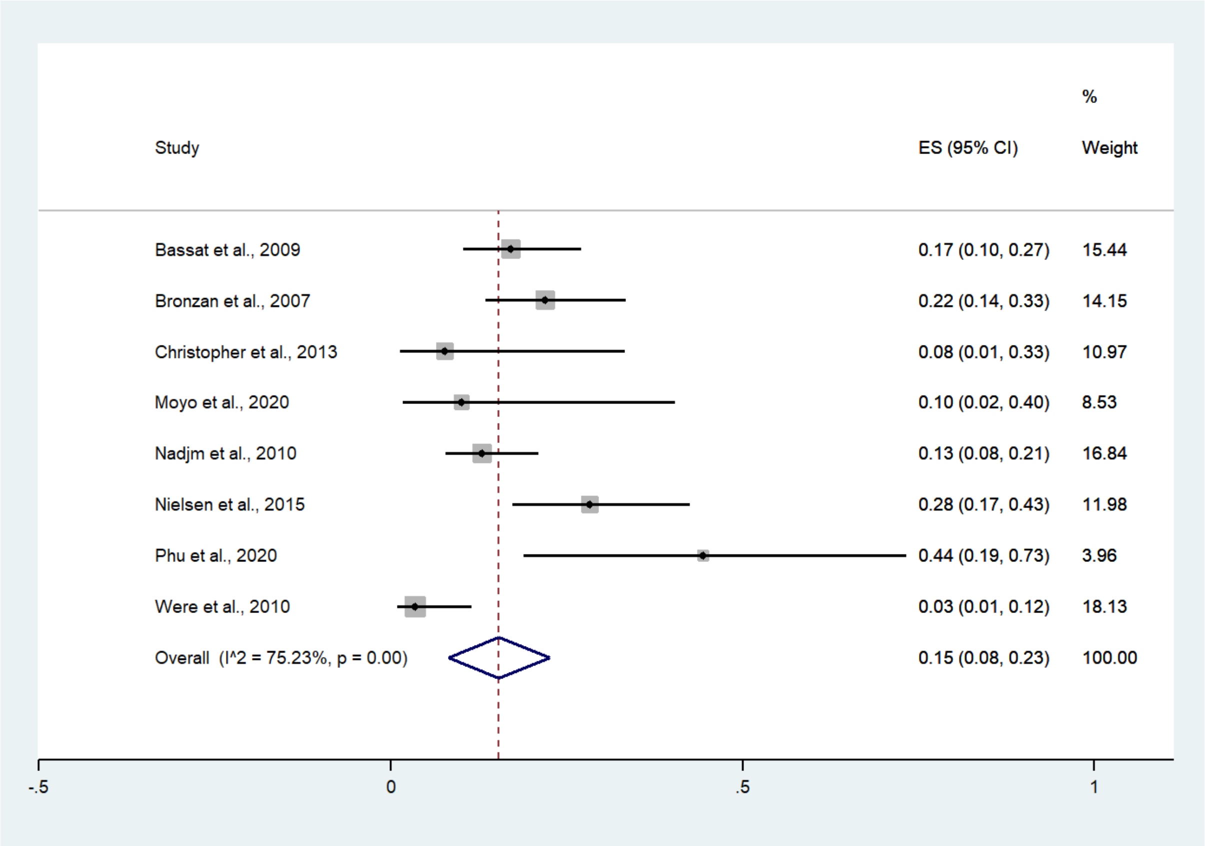

Supplementary Figure S13. The pooled prevalence of deaths among patients with co-infection. Abbreviations: ES: prevalence estimate; CI, confidence interval

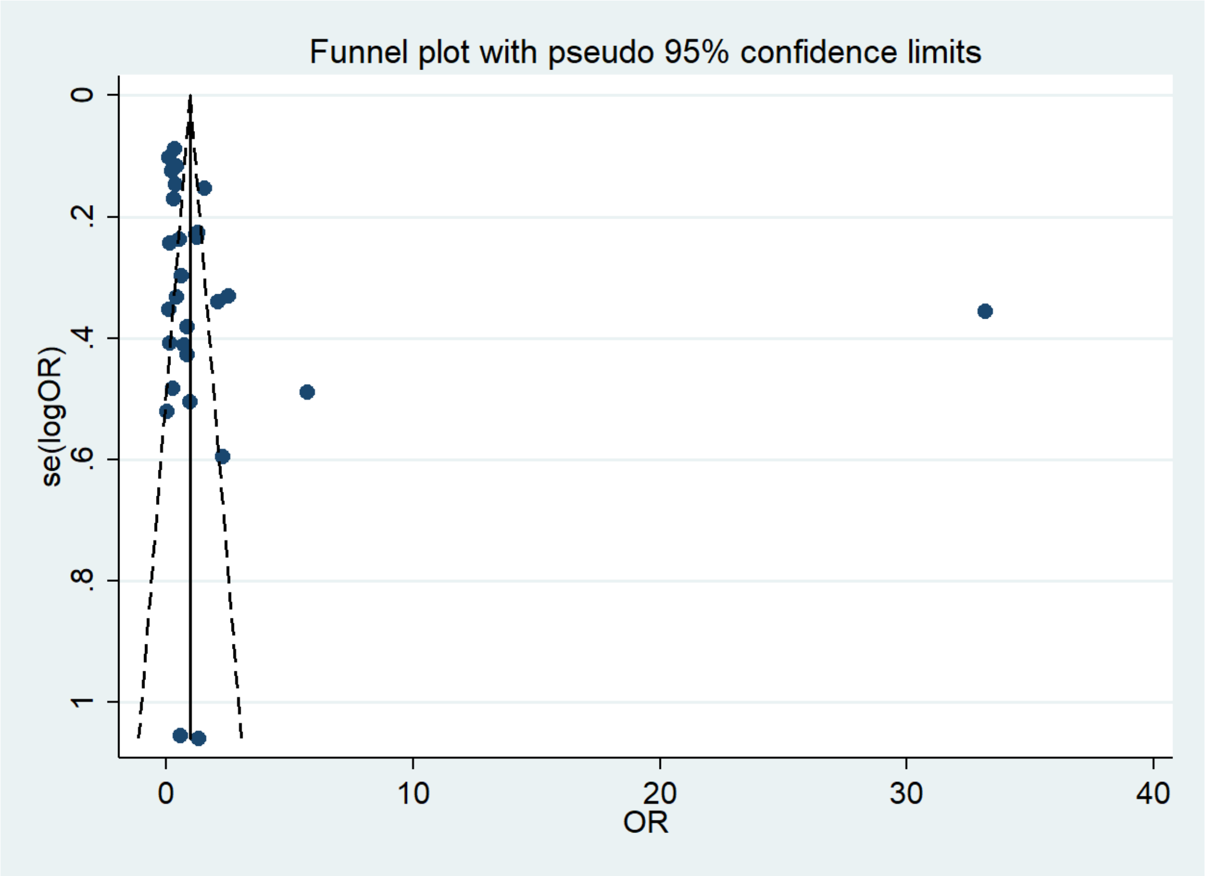

Supplementary Figure S14. A funnel plot of the probability of co-infection.
