# Supplementary material for: The Prevalence of Malaria and Bacteremia Co-Infections among Febrile Patients: A Systematic Review and Meta-Analysis
Source: Trop Med Infect Dis. 2022 Sep 13;7(9):243. doi: 10.3390/tropicalmed7090243 (PMC9503679; doi:10.3390/tropicalmed7090243)
Supplement: Supplementary file 1 [file tropicalmed-07-00243-s001.zip › Table S1. Search terms.pdf]

Table S1. Search terms

**Databases**	**Search terms/Search strategy**	**Date**
MEDLINE	(malaria OR plasmodium) AND (bacteremia)<br>Search results: 288	13 September 2021
Scopus	(malaria OR plasmodium) AND (bacteremia)<br>Search option: Title, abstract, keywords<br>Search results: 417	13 September 2021
Web of Science	(malaria OR plasmodium) AND (bacteremia)<br>Search option: All fields<br>Search results: 337	13 September 2021
