# Supplementary material for: The Prevalence of Malaria and Bacteremia Co-Infections among Febrile Patients: A Systematic Review and Meta-Analysis
Source: Trop Med Infect Dis. 2022 Sep 13;7(9):243. doi: 10.3390/tropicalmed7090243 (PMC9503679; doi:10.3390/tropicalmed7090243)
Supplement: Supplementary file 1 [file tropicalmed-07-00243-s001.zip › Table S2. Details of the included studies.pdf]

Table S2. Details of the included studies

Author	Study site	Year conducted	Study design	Types of participants enrolled	Number of participants	Age range	%Male	Number of malaria and bacteremia co-infection	All co-infection	All malaria	All bacteremia	Malaria without bacteremia	Bacteremia without malaria	Non-malaria/ bacteremia	Clinical outcomes of co-infection	Test for malaria	Test for bacteremia
Akinyemi et al., 2007	Nigeria	2004-2005	Cross-sectional study	Febrile	441	All age groups	NS	*Salmonella* (16), Without *Salmonella* (91)	16	107	42	91	26	308	NS	Thick-blood smear	Blood culture
Akpede et al., 1993	Nigeria	1988-1989	Prospective observational study	Convulsion associated with fever	386	All age groups	NS	*P. falciparum* + gram positive bacteria (9), *P. falciparum* + gram negative bacteria (18)	27	289	43	262	16	81	NS	Blood film examination	Blood culture
Archibald et al., 1998	Tanzania	1995	Prospective observational study	Febrile	517	≥15 years old	280 (54.2%)	Malaria + Bacteremia unidentified (5)	5	49	145	44	140	328	NS	Thin-blood smear	Blood culture
Archibald et al., 2003	Malawi	1998	Cross-sectional study	Pediatric patients had initiated antimicrobial, antituberculous, or antimalarial therapy	229	All age groups	128 (55.9%)	Malaria + Bacteremia unidentified (2)	2	2	35	0	33	194	NS	Thick and thin-blood smear	Blood culture
Aung et al., 2018	Myanmar	2016-2017	Prospective cohort study	Patients with falciparum malaria	20	≥ 16 years old	19 (95%)	*P. falciparum* + *Escherichia coli* (1), *Salmonella typhi* (1), *Staphylococcus aureus* (1), mixed growth of *Acinetobacter baumannii* and *Enterobacter cloacae* (1)	4	20	4	16	0	0	NS	thick and thin-blood film examination	Blood culture
Ayoola et al., 2005	Nigeria	NS	Prospective observational study	Febrile	102	Infants aged 1 to 12 months	NS	*P. falciparum* + gram positive bacteria (9), *P. falciparum* + gram negative bacteria (7)	16	47	39	31	23	32	Diarrhea and restlessness	Thick and thin-blood film examination	Blood culture
Bassat et al., 2009	Mozambique	2003-2007	Retrospective study	Children with severe malaria	1780	Children < 5 years old	NS	*P. falciparum* + *S. pneumoniae* (20), +gram negative bacteria (16), +*S. aureus* (13) and +NTS (12)	76	1780	76	1,704	0	0	Higher case-fatality rates	Thick and thin-blood film examination	Blood culture
Bell et al., 2001	Malawi	1998	Prospective observational study	Febrile	238	> 14 years old	101 (42.4%)	Malaria + gram negative bacteria (21), Malaria +*S. pneumoniae* (1)	22	64	52	42	30	144	NS	Thick-blood smear	Blood culture
Bhattacharya et al., 2013	India	2004	Prospective observational study	Febrile	3,371	All age groups	1,730 (51.3%)	*P. vivax*+ S. Typhi (1), +*S. paratyphi A* (1), other gram negative bacteria (3), *P. vivax* + gram positive bacteria (1)	6	93	256	87	250	3,028	Increase vivax relapses	Thin-blood film examination	Blood culture
Biggs et al., 2013	Tanzania	2006-2008	Prospective observational study	Febrile	4,106	2 months to 13 years	2,237 (54.5%)	Malaria (*P. falciparum*) + Nontyphoidal *Salmonella* (NTS) (53), recent malaria (*P. falciparum*) + Non-typhoidal *Salmonella* (NTS) (67)	120	2206	356	2086	236	1,664	NS	Microscopy, rapid diagnostic test	Blood culture
Blomberg et al., 2007	Tanzania	2001-2002	Prospective cohort study	Children with signs of systemic infection	1,787	Children 0 to 7 years (median age of 8.5 months)	992 (56%)	Malaria + Bacteremia unidentified (18)	18	49	62	21	44	1,704	Higher case-fatality rates	Thick and thin drop blood smears	Blood culture

Author	Study site	Year conducted	Study design	Types of participants enrolled	Number of participants	Age range	%Male	Number of malaria and bacteremia co-infection	All co-infection	All malaria	All bacteremia	Malaria without bacteremia	Bacteremia without malaria	Non-malaria/ bacteremia	Clinical outcomes of co-infection	Test for malaria	Test for bacteremia
Boumbanda Koyo et al., 2020	Gabon	2015-2016	Retrospective study	Febrile	428	< 15 years old	NS	*P. falciparum* + *H. influenzae*, S. aureus, and *S. pneumoniae* (1), *P. falciparum* +*P. malariae* +*H. influenzae* (1), *P. falciparum* +*Rickettsia felis* (4), *P. falciparum* +*S. pneumoniae* (3), *P. falciparum* +*S. aureus* (3), *P. falciparum* +*H. influenzae* (1)	13	298	19	285	6	124	NS	RT-PCR, PCR	RT-PCR, PCR
Bronzan et al., 2007	Malawi	1996-2005	Prospective observational study	Children with severe malaria	1,388	Children ≥6 months old	710 (51.2%)	*P. falciparum* +Non-typhoidal *Salmonella* (37), +*Streptococcus pneumoniae* (7), +*Klebsiella* species (3), +*Acinetobacter* species (3), +alpha-hemolytic *Streptococcus* (2), +*Salmonella typhi* (1), +*Aeromonas* species (1), +*Escherichia vulneris/Proteus mirabilis* (1), +*Staphylococcus aureus* (1), +group D *Streptococcus* (1), +gram-negative rods (1), +*Haemophilus influenzae* type b (1), +non-typeable *Haemophilus influenzae* (1), +*Escherichia coli* (1), +*Campylobacter* species (1), +*Enterobacter cloacae* (1), and *enterococcus* (1)	64	1,388	64	1,324	0	0	Higher case-fatality rates	Blood film examination	Blood culture
Christopher et al., 2013	Tanzania	2011-2012	Prospective cross-sectional study	Febrile	317	2 to 60 months old	195 (61.5%)	Malaria +Bacteremia unidentified (13)	13	82	21	69	7	212	High mortality rates	Blood slide	Blood culture
Egbe et al., 2014	Nigeria	NS	Cross-sectional study	Febrile	500	All age groups (1 day to 84 years)	281 (56.2%)	Malaria +Bacteremia unidentified (39)	39	170	104	131	65	265	NS	Thick-blood film examination	Blood culture
Ehounoud et al., 2021	the Republic of Cˆote d'Ivoire (Ivory Coast)	2014-2015	Cross-sectional study	Febrile	438	All age groups	176 (40.3%)	*P. falciparum* +*P. malariae* +*S.pneumoniae* (1), *P. falciparum* +*P. ovale* +*R. felis* (1), *P. falciparum* +*P. ovale* +*S. aureus* (2), *P. falciparum* +*R. felis* +*S. aureus* (1), *P.falciparum*+*S. aureus*+*S. pneumoniae* (2), P. *ovale*+*S. aureus*+*S. enterica Typhi* (1), *P. falciparum*+*R. felis* (8), *P. falciparum*+*S. aureus* (10), *P.falciparum*+*Non-typhoidal Salmonella* (10)	36	217	95	181	59	162	NS	RT-PCR	RT-PCR
Ekanem et al., 2008	Nigeria	2000-2002	Prospective observational study	Neonates with features suggestive of sepsis	202	Neonates ≤ 1 week old	112(55.4%)	*P. falciparum* +*Staphylococcus aureus* (6), +coliform (6) and + others/unidentified (2)	14	71	103	57	89	42	NS	Thick and thin-blood film examination	Blood culture

Author	Study site	Year conducted	Study design	Types of participants enrolled	Number of participants	Age range	%Male	Number of malaria and bacteremia co-infection	All co-infection	All malaria	All bacteremia	Malaria without bacteremia	Bacteremia without malaria	Non-malaria/ bacteremia	Clinical outcomes of co-infection	Test for malaria	Test for bacteremia
Evans et al., 2004	Ghana	NS	Retrospective study /Prospective observational study	Children with severe malaria	251	NS	127 (50.5%)	*P. falciparum* +Non-typhoid *Salmonella* species (NTS) (10), +*Staphylococcus aureus* (9), + *Escherichia coli* (1), +*Providencia* (1), +Other coliforms (1), +*Streptococcus pneumoniae* (1)	23	182	51	159	28	41	NS	Thick and thin-blood film examination	Blood culture
Ganiyu et al., 2004	Nigeria	NS	Cross-sectional study	Children	147	4.24 ± 2.88 years of age	NS	Malaria +*E. coli* (5), +*Proteus* (4), +*Staphylococcus* (16), +*Salmonella* (1)	26	60	28	34	2	85	NS	Thick and thin-blood film examination	Blood culture
Graham et al., 2002	Malawi	1996-1999	Prospective observational study	Children with severe malaria	701	3-148 months	NS	Malaria +NTS (18), Malaria +*Streptococcus pneumoniae* (5), + Group D *Streptococcus* (1), +alpha-haemolytic *Streptococcus* (1) and *Enterococcus* (1); +Enterobacteriacere were *Enterobacter cloacae* (2), *Acinetobacter* (2), *Escherichia coti* (1), *Salmonella typhi* (1), and *Klebsiella* (1), +*Campylobacter* spp. (1), *Haemophilus influenzae* (1)	36	701	36	665	0	0	NS	Thin and thick film microscopy	Blood culture
Guiraud et al., 2017	Burkina Faso	2013-2014	Prospective cohort study	Febrile	1,339	2 months-15 years old	NS	*P. falciparum* +iNTS (12), +*Salmonella Typhi* (3), +*Streptococcus pneumoniae* (5), +*Staphylococcus aureus* (2)	22	777	118	755	96	466	NS	Thick-blood film examination	Blood culture
Hogan et al., 2018	Ghana	2013-1015	Prospective cohort study	Febrile	1,238	≥30 days and ≤15 years	677 (55%)	Malaria +Bacteremia unidentified (4)	4	728	62	724	58	452	NS	Microscopy	Blood culture
Ikumapayi et al., 2007	Gambia	2002-2004	Cross-sectional study	Children who were investigated as possible cases of invasive bacterial infection	62	Children aged 2–29 months	NS	Malaria +Bacteremia unidentified (25)	25	0	62	0	37	0	NS	NS	Blood culture
Isendahl et al., 2014	Guinea-Bissau	2010	Cross-sectional study	Children with tachycardia and/or fever	372	NS (mean age was 1.7 years)	209 (56%)	Malaria +Bacteremia unidentified (4)	4	17	46	13	42	313	NS	Thin and thick smears for malaria microscopy	Blood culture
Kabore et al., 2021	Burkina Faso	2013-2014	Prospective observational study	febrile	589	children aged 3 months to 15 years	350 (59%)	*P. falciparum* +Bacteremia (16)	16	175	75	159	59	355	NS	Microscopy , rapid diagnostic test [RDT] and PCR	Blood culture, PCR
Krumkamp et al., 2016	Ghana	2007-2012	Case-control study	Febrile	6,746	Children <15 years of age	3,661 (54.3%)	*P. falciparum* +Invasive Non-typhoidal *Salmonella* (34)	34	2,530	445	2,479	411	3,822	NS	Thick-blood film examination	Blood culture

Author	Study site	Year conducted	Study design	Types of participants enrolled	Number of participants	Age range	%Male	Number of malaria and bacteremia co-infection	All co-infection	All malaria	All bacteremia	Malaria without bacteremia	Bacteremia without malaria	Non-malaria/ bacteremia	Clinical outcomes of co-infection	Test for malaria	Test for bacteremia
Lepage et al., 1987	Rwanda	1984-1985	Case-control study	Febrile	900	Children aged under 15 years	NS	*P. falciparum* +Bacteremia (30)	30	30	112	0	82	788	NS	Thick-drop smear	Blood culture
Maltha et al., 2014	Burkina Faso	2012-2013	Prospective observational study	Febrile and/or signs of severe illness	711	children aged under 15 years	393 (55.3%)	Malaria +Bacteremia unidentified (7)	7	292	63	285	56	363	Higher case-fatality rates	Thick and thin-blood film examination , rapid diagnostic test [RDT]	Blood culture
Morch et al., 2017	India	2011-2012	Prospective observational study	Febrile	1,564	≥5 years old	895 (57%)	*P. falciparum* (12), other *Plasmodium* (13) +Bacteremia include , *Staphylococcus aureus, Salmonella typhi* and *S. paratyphi*	25	268	124	243	99	1,197	NS	Blood smear, rapid diagnostic test [RDT] and PCR	Blood culture
Mourembou et al., 2016	Gabon	NS	Case-control study	Febrile	410	Children under 15 years	212 (51.7%)	*P. falciparum +T. whipplei, S. aureus, and S. pneumoniae* (1), *P. falciparum +S. aureus* and *S. pneumoniae* (3), *P. falciparum +S. aureus,* and *S. pyogenes* (1), *P. falciparum+non-typhi Salmonella* (3), *P. falciparum +S. aureus* (1), *P. falciparum +S. pneumoniae* (5)	14	323	22	309	8	79	NS	Thick blood film, RT-PCR	blood cultures, RT-PCR
Moyo et al., 2020	Tanzania	2017-2018	Cross-sectional study	Febrile	2,226	Children aged 0–5 years	1,299 (58%)	*P. falciparum +Serratia marcescens* (2), +*K. pneumoniae (*2), +*E. coli* (1), +*Acinetobacter ursingii* (1), +*Alcaligenes faecalis* (1), +*Ochrobactrum tritici* (1), *E. faecium* (1), and *S. aureus* (1)	10	204	236	194	226	1,796	40% (4/10) had clinically severe disease and high malaria parasitaemia by qPCR, and one of these four children died.	RT-PCR	Blood culture
Msaki et al., 2012	Tanzania	2011	Cross-sectional study	Febrile	231	Children less than 5 years of age	105 (45.5%)	*P. falciparum* malaria +bacteremia (1)	1	22	17	21	16	193	NS	Thin and thick blood smears	Blood culture
Mtove et al., 2011	Tanzania	2006-2010	Prospective observational study	Febrile	6,836	Children aged 2 months to 14 years	2,422 (46%)	*P. falciparum* malaria +bacteremia (167)	167	1,898	1,248	1,731	1,081	3,857	NS	Thick and thin-blood film examination , rapid diagnostic test [RDT]	Blood culture
Muthumbi et al., 2015	Kenya	1998-2014	Retrospective study	Febrile or signs of focal sepsis	98,600	All age groups	52,341 (53.1%)	*P. falciparum* malaria + NTS bacteremia (99)	99	18,896	3,814	18,797	3,715	75,989	NS	Thick and thin films	Blood culture
Nadjm et al., 2010	Tanzania	2006-2007	Prospective observational study	Febrile	3,639	Children aged 2 months to 13 years	NS	P. falciparum + NTS (52), P. falciparum+ other bacteria (48)	100	2,195	341	2,095	241	1,203	higher case-fatality rates	Microscopy , rapid diagnostic test [RDT]	Blood culture

Author	Study site	Year conducted	Study design	Types of participants enrolled	Number of participants	Age range	%Male	Number of malaria and bacteremia co-infection	All co-infection	All malaria	All bacteremia	Malaria without bacteremia	Bacteremia without malaria	Non-malaria/ bacteremia	Clinical outcomes of co-infection	Test for malaria	Test for bacteremia
Nielsen et al., 2015	Ghana	2007-2011	Prospective observational study	severe illness	1,915	Children under 15 years	55%	*P. falciparum* +NTS (21), +*Streptococcus* spp. (6), +*S. aureus* (4), +*S. pneumoniae* (3), +*Pseudomonas* spp. (3), +*Salmonella ser. Typhi* (2), +*Escherichia coli* (2), +*Acinetobacter* spp. (2), +*Klebsiella* spp. (1), +*Shigella* spp. (1) and *Chryseobacter* spp. (1)	46	771	244	725	198	946	higher case-fatality rates	Thin and thick blood smears	Blood culture
Nyein et al., 2016	Myanmar	2014-2015	Cross-sectional study	Adults with falciparum malaria	67	Adults (NS)	54 (80.6%)	*P. falciparum* +*Escherichia coli* (3), +*Salmonella* Paratyphi A (2), *Staphylococcus aureus* (2), +*Salmonella typhi* (1) and +*Salmonella typhimurium* (1)	9	67	9	58	0	0	NS	Rapid diagnostic test [RDT]	Blood culture
Okwara et al., 2004	Kenya	2001	Cross-sectional study	Febrile	264	Children aged between three months and 12 years	156 (59.1%)	*P. falciparum* +bacteremia (18)	18	158	32	140	14	92	NS	Thick-blood film examination	Blood culture
Onchiri et al., 2015	Kenya	2012-2014	Prospective cohort study	Febrile	1,476	Children aged 6 months to 15 years	701 (47.5%)	Malaria+Bacteremia unidentified (8)	8	428	48	420	40		NS	Thin and thick smear microscopy, rapid diagnostic tests (RDTs)	Blood culture
Park et al., 2016	Sub-Saharan Africa (Burkina Faso, Ethiopia, Ghana, Guinea-Bissau, Kenya, Madagascar, Senegal, South Africa, Sudan, and Tanzania)	2010-2014	Cross-sectional study	Febrile	13,431	All age groups (except in Ghana, where where only children aged <15 years	NS	Malaria +gram-positive bacteria (22), Mararia +NTS (15), Malaria + *Salmonella* Typhi (9), Malaria + gram negative bacteria (7)	53	3,133	497	3,080	444	9,854	NS	Thick and thin blood smears and rapid malaria tests	Blood culture

Author	Study site	Year conducted	Study design	Types of participants enrolled	Number of participants	Age range	%Male	Number of malaria and bacteremia co-infection	All co-infection	All malaria	All bacteremia	Malaria without bacteremia	Bacteremia without malaria	Non-malaria/ bacteremia	Clinical outcomes of co-infection	Test for malaria	Test for bacteremia
Pattanaik et al., 2012	India	2008-2009	Prospective cohort study	Patients categorized into severe and uncomplicated malaria	67	Adults (NS)	NS	*P. falciparum* +*Streptococcus pyogenes* (1)	1	67	1	66	0	0	NS	Thick and thin blood smears, rapid diagnostic test [RDT], nested polymerase chain reaction (PCR)	Blood culture
Phoba et al., 2013	Democratic Republic of the Congo (DRC)	2011-2012	Prospective observational study	Febrile	3,311	Children < 5 years old	NS	*P. falciparum* +*Salmonella* spp. (74), *P. falciparum* + other (7)	81	184	114	103	33		NS	Thick and thin blood smears, rapid diagnostic test [RDT]	Blood culture
Phu et al., 2020	Vietnam	1991-2003	Prospective observational study	Adults with severe falciparum malaria	845	Older than 14 years	NS	*P. falciparum* +S. Typhi (3), +non-typhoid *Salmonella* (1), +*Staphylococcus aureus* (2), +Group A *Streptococcus* (1), *H. influenzae* type b (1), and *Klebsiella pneumoniae* (1)	9	845	9	836	0	0	Higher case-fatality rates	Thin- and thick-film malaria parasite counts	Blood culture
Popoora et al., 2019	Nigeria	2017	Cross-sectional study	Febrile	682	All age groups	332 (48.7)	*P. falciparum* +*S.* Typhi (6), +non-typhoid *Salmonella* (1), +*Staphylococcus aureus* (24), + *Klebsiella pneumoniae* (1), +*Citrobacter koseri* (1) and *Enterobacter cloacae* (1)	34	171	117	137	83	428	NS	Microscopy of thin and thick films	blood cultures, PCR
Sandlund et al., 2012	Sweden	1995-2009	Retrospective study	travelers diagnosed with malaria	755	All age groups	NS	*P. falciparum* +*Salmonella* enterica serovar Enteritidis (1), *Plasmodium* spp. + *Escherichia coli* (1)	2	755	2	753	0	0	NS	Thick and thin blood smears, rapid diagnostic test [RDT], PCR	Blood culture
Shimelis et al., 2020	Ethiopia	2018-2019	Prospective cross-sectional study	Febrile	433	Children aged at least 2 months and under13 years	255 (58.9%)	Malaria +Bacteremia unidentified (1)	1	14	24	13	23	396	NS	Thick and thin blood smear slides	Blood culture
Sothmann et al., 2015	Ghana	2012	Case-Control Study	Febrile	2,306	Children below 15 years of age	1235 (53.6%)	Malaria +Bacteremia unidentified (16)	16	866	72	850	56	1,384	NS	Thin and thick blood slides	Blood culture
Thriemer et al., 2012	Tanzania	2009-2010	Case-Control Study	Febrile	2,209	Above the age of 2 months	NS	*P. falciparum* + bacteremia (1)	1	29	166	28	165	2,015	NS	Thin and thick blood films	Blood culture

Author	Study site	Year conducted	Study design	Types of participants enrolled	Number of participants	Age range	%Male	Number of malaria and bacteremia co-infection	All co-infection	All malaria	All bacteremia	Malaria without bacteremia	Bacteremia without malaria	Non-malaria/ bacteremia	Clinical outcomes of co-infection	Test for malaria	Test for bacteremia
Ukaga et al., 2006	Nigeria	NS	Case-Control Study	Malaria positive and apparently healthy patients	185	All age groups	NS	*Staphylococcus aureus* 4 (3.2%), *Escherichia coli* 3 (2.4%) *Salmonella typhi* 25 (20%), *Klebsiella pneumoniae* 10 (2.4%) and *Pseudomonas aeruginosa* 2 (1.6%).	44	125	44	81	0	60	Persistence of malaria-like symptoms after treatment	Thick and thin blood smears s	Blood culture
Walsh et al., 2000	Malawi	1996-1997	Prospective observational study	Febrile	2,123	Neonate or child >2 months of age	NS	*P. falciparum* + bacteremia (67)	67	290	365	223	298	1,535	Splenomegaly and anemia	Blood film examination	Blood culture
Were et al., 2010	Kenya	2004-2006	Prospective cohort study	*P. falciparum* malaria	585	Aged 1 to 36 months	295 (50.4%)	*P. falciparum* + gram positive bacteria (22), + gram-negative bacteria (37)	59	585	59	526	0	0	Higher case-fatality rates	Thick and thin blood films	Blood culture
